# 16S rRNA sequence embeddings: Meaningful numeric feature representations of nucleotide sequences that are convenient for downstream analyses

**DOI:** 10.1371/journal.pcbi.1006721

**Published:** 2019-02-26

**Authors:** Stephen Woloszynek, Zhengqiao Zhao, Jian Chen, Gail L. Rosen

**Affiliations:** 1 Department of Electrical and Computer Engineering, Drexel University, Philadelphia, Pennsylvania, United States of America; 2 Department of Computer Science and Engineering, State University of New York at Buffalo, Buffalo, New York, United States of America; DAL, CANADA

## Abstract

Advances in high-throughput sequencing have increased the availability of microbiome sequencing data that can be exploited to characterize microbiome community structure *in situ*. We explore using word and sentence embedding approaches for nucleotide sequences since they may be a suitable numerical representation for downstream machine learning applications (especially deep learning). This work involves first encoding (“embedding”) each sequence into a dense, low-dimensional, numeric vector space. Here, we use Skip-Gram word2vec to embed *k*-mers, obtained from 16S rRNA amplicon surveys, and then leverage an existing sentence embedding technique to embed all sequences belonging to specific body sites or samples. We demonstrate that these representations are meaningful, and hence the embedding space can be exploited as a form of feature extraction for exploratory analysis. We show that sequence embeddings preserve relevant information about the sequencing data such as *k*-mer context, sequence taxonomy, and sample class. Specifically, the sequence embedding space resolved differences among phyla, as well as differences among genera within the same family. Distances between sequence embeddings had similar qualities to distances between alignment identities, and embedding multiple sequences can be thought of as generating a consensus sequence. In addition, embeddings are versatile features that can be used for many downstream tasks, such as taxonomic and sample classification. Using sample embeddings for body site classification resulted in negligible performance loss compared to using OTU abundance data, and clustering embeddings yielded high fidelity species clusters. Lastly, the *k*-mer embedding space captured distinct *k*-mer profiles that mapped to specific regions of the 16S rRNA gene and corresponded with particular body sites. Together, our results show that embedding sequences results in meaningful representations that can be used for exploratory analyses or for downstream machine learning applications that require numeric data. Moreover, because the embeddings are trained in an unsupervised manner, unlabeled data can be embedded and used to bolster supervised machine learning tasks.

## Author summary

Improvements in the way genomes are sequenced have led to an abundance of microbiome data. With the right approaches, researchers use these data to thoroughly characterize how microbes interact with each other and their host, but sequencing data is of a form (sequences of letters) not ideal for many data analysis approaches. We therefore present an approach to transform sequencing data into arrays of numbers that can capture interesting qualities of the data at the sub-sequence, full-sequence, and sample levels. This allows us to measure the importance of certain microbial sequences with respect to the type of microbe and the condition of the host. Also, representing sequences in this way improves our ability to use other complicated modeling approaches. Using microbiome data from human samples, we show that our numeric representations captured differences between various types of microbes, as well as differences in the body site location from which the samples were collected.

This is a *PLoS Computational Biology* Methods paper.

## Introduction

Recent advances in high-throughput sequencing techniques have dramatically increased the availability of microbiome sequencing data, allowing investigators to identify genomic differences among microbes, as well as characterize microbiome community structure *in situ*. A microbiome is defined as the collection of microorganisms (bacterial, archaeal, eukaryotic, and viral) that inhabit an environment. Recent work has shown that there exists reciprocal interplay between microbiome and environment, such that the configuration of microbes is often influenced by shifts in environmental state (such as disease in human hosts [1–3], chemical alterations in soil [4, 5], or oceanic temperature changes [6]). Conversely, the environmental state may be impacted by particular microbial profiles [7–10].

Identifying and characterizing important microbial profiles often entails sequencing collections of heterogeneous, fragmented genomic material, which act as a proxy for the microbiome’s configuration *in situ*. Sequencing these fragments yields short strings of nucleotides (“sequence reads”) with no easily discernible clues to determine from which microbe they originated. Still, the order of nucleotides within each sequence provides enough information for approaches that utilize sequence alignment or sequence denoising algorithms, along with taxonomic reference databases, to quantify the abundance of sequences belonging to different microbes at different taxonomic levels (*e.g*., genus), which in turn can be associated with environmental factors [11, 12]. Thus, a given sample is represented as a vector of hundreds, often thousands, taxonomic counts (nonnegative integers), where the taxa have been traditionally termed Operational Taxonomic Units (OTUs). Clustering sequences into OTUs that approximate species has relied on the 97% similarity heuristic, which has been widely criticized as a poor threshold for hypervariable regions and as not being biologically meaningful [13–16] and can vary from lineage to lineage [17]. Moreover, in Callahan *et al*. [14], the authors argue that even though clustering OTUs helps diminish the influence of Illumina sequencing errors, it nevertheless discards many of the subtle differences between sequences. Other work has shown that reference gene methods poorly reflect community diversity because they depend on the database used in the analysis [18–20] Given this, alternative approaches to alignment-based similarity, especially those that can take into account subfeatures and their context, warrant exploration.

An alternative approach to aligning or denoising nucleotide sequences is to represent the nucleotide sequences numerically. One can then search for similarities among these numeric features. In addition, these numeric representations are more suitable for machine learning algorithms. Two examples of such approaches are one-hot-encoding and *k*-mer (n-gram) counting [21, 22]. With one-hot-encoding (also referred to as generating binary indicator sequences [23]), each sequence is binarized—that is, each nucleotide (ACGT) is represented as a unit vector of length four, with a value of one indicating the presence of a particular nucleotide. *k*-mer counting, on the other hand, counts the frequency of all possible substrings of length *k* in a sequence. A larger *k* yields a higher dimensional, more sparse representation of the sequence since there are more possible *k*-mers (4^k^) (“the curse of dimensionality” [21, 24]), but a smaller *k* is unlikely to capture much of the nucleotide-to-nucleotide sequential variation among sequences [25]. With the large GreenGenes database [26], there are over 2 million sequences comprising over 2.5 billion basepairs, and therefore using *k*-mers up to 15 (4^15^ features) reduces the basepairs to a lower dimensional representation. However, sample classification usually uses OTUs which are on the order of tens of thousands. Thus, if *k*-mers are to be used and lower dimensionality is desired, a representation that further reduces the dimensions of the *k*-mers is needed. Either set of engineered features (one-hot-encodings or *k*-mer frequencies) can be used in various machine learning algorithms to characterize the sequences in some way [27–30]. Still, one-hot-encoding or *k*-mer frequencies, when *k* is large, yield sparse, high-dimensional features that often present difficulties during training [31]. In addition, neither approach encodes the relative ordering of the *k*-mers [21, 32].

A more suitable representation of nucleotide sequences involves first encoding (“embedding”) each sequence into a dense, numeric vector space via the use of word embedding algorithms such as word2vec [27]. Word embeddings are commonly used for natural language processing [27, 33–36]. Various architectures exist, but their objective is generally the same: capture semantic and lexical information of each word based on that word’s context—*i.e*., its neighboring set of words. Each word is represented in a vector space of predefined length, where semantically similar words are placed near one another. Thus, *k*-mer representations of sequences could be embedded in such a way that their context is preserved (the position of *k*-mers relative to their neighbors), and they become suitable for down-stream machine learning approaches. Recent work has successfully embedded short, variable-length DNA *k*-mers [21], as well as protein sequences for down-stream tasks such as protein structure prediction [37]. Ng [21] showed that the cosine similarity between embedded *k*-mers is positively correlated with Needleman-Wunsch scores obtained via global sequence alignment. In addition, he showed that vector arithmetic of two *k*-mer embeddings is analogous to concatenating their nucleotide sequences. This finding is consistent with work demonstrating the ability of vector arithmetic to solve word analogies, such as “King is to Queen, as Man is to _____” [38].

Thus, here we explore word embeddings as a means to represent 16S rRNA amplicon sequences, obtained from microbiome samples, as dense, low-dimensional features that preserve *k*-mer context (*i.e*., leverage the relative position of *k*-mers to their neighbors). We use Skip-Gram word2vec to perform the initial *k*-mer embedding. Then, we leverage an existing sentence embedding technique [39] to embed individual nucleotide sequences or sets of sequences (*e.g*., all sequences belonging to a given sample) from *k*-mer embeddings. This sentence-embedding technique interestingly does not explicitly encode word order; yet, it has shown to outperform competing methods such as recurrent neural networks in textual similarity tasks [39].

The sentence embedding procedure is simple, but effective, consisting of down-weighting the embeddings for high frequency *k*-mers, averaging the *k*-mer embeddings that constitute a given sequence or set of sequences (forming a sequence or set embedding), and then subtracting the projection of the sequence/set embedding to its first principal component (“common component removal”, which, per [39], we refer to as “denoising”). Representing nucleotide sequences in vector space provides multiple benefits that may prove valuable in characterizing a microbiome: (1) the embeddings are dense, continuous, and relatively low-dimensional (compared to using *k*-mer frequencies, for example), making them suitable for various down-stream machine learning tasks; (2) they leverage *k*-mer context, yielding potentially superior feature representations compared to *k*-mer frequencies; (3) once trained, the *k*-mer-to-embedding mapping vectors can be stored and used to embed any set of 16S rRNA amplicon sequences; (4) the embedding model can be trained with data that are independent of the query sequences of interest, such that the training procedure can leverage a significant amount of unlabeled data that would otherwise go unused; (5) feature extraction is performed at the sequence level, enabling one to detect relationships between sample-level information (*e.g*., soil quality) and all sequences belonging to a given sample and then traceback to determine not only which sequences are key, but also which *k*-mers; and (6) once important *k*-mers are identified, because the embedding initially takes place at the *k*-mer level, the *k*-mer contextual information is available, which may indicate the neighborhood noteworthy *k*-mers occupy.

In this work, we prove that the embedding space performs well at classifying samples, predicting the correct sample class (*e.g*., body site) given the embedding of all its sequences. Moreover, because these embeddings are encoded from *k*-mer embeddings, their classification performance helps justify the use of *k*-mer embeddings as input in more complex architectures such as deep neural networks. We show that the embedding space provides a set of meaningful features that capture sample-level (sample class), taxonomic-level (sequence, read), and sequence-level (*k*-mer, *k*-mer context) characteristics, which not only justifies the use of the embedding space for supervised tasks such as classification, but also justifies its use for unsupervised feature extraction, to capture meaningful signal for the exploratory phase of a given analysis. Lastly, we illustrate approaches that may help disentangle what the embedding learned from the data, in the context of microbiome information, such as taxonomy and sample information.

## Results and discussion

We will use the following terminology for the remaining sections. “Query” sequences and *k*-mers are all sequences and *k*-mers that were not used for training (and hence are not in the GreenGenes reference database). A “*k*-mer” embedding is an d-dimensional vector containing the embedding of a nucleotide subsequence of length *k*. A “sequence embedding” is a d-dimensional vector formed by performing the sentence embedding approach by [39] on all *k*-mers belonging to a single nucleotide sequence (*e.g*., a 16S rRNA full-length sequence or read). “Cluster embeddings” or “sample embeddings” are also d-dimensional vectors, but they encode an embedding that includes all *k*-mers belonging to a set of sequences (such as a set identified via clustering) or a set that includes all sequences belonging to a single sample, respectively. Lastly, “denoising” refers to common-component removal (not dada2-style “sequence denoising algorithms”), which is performed when we apply the sentence embedding approach to sequences, sequence reads, clusters of sequences, or all sequences belonging to specific samples.

### Evaluation of sequence embeddings on full-length 16S rRNA amplicon sequences

We began by evaluating the performance of sequence embeddings. DNA alignment combined with clustering [11] or sequencing denoising algorithms [12] are readily capable of identifying sequence-level differences between genera. We consequently aimed to discern if genus-level differences were in fact detectable in the sequence embedding vector space. Genus level resolution is not only relevant to characterizing a microbial community; it also would suggest that the vector space is capable of capturing subtle, but important sequence differences among taxa. These differences may be critical in characterizing data at the sample level, particularly during classification, where its desired to discern how the configuration of microbes comprising a sample (*i.e*., all its sequences) is influenced by sample-level information such as soil quality.

The *k*-mer embedding space was obtained by training Skip-Gram word2vec on 2,262,986 full-length 16S rRNA amplicon sequences from the GreenGenes reference database (Fig B in S1 Appendix). An independent set of 16,699 16S rRNA sequences from the KEGG REST server [40] were obtained as our test dataset. (We will refer to these sequences as “KEGG 16S sequences” henceforth.) 14,520 contained *k*-mers that intersected with the training set and thus were sequence-embedded using the sentence embedding approach by [39]. Briefly, for each sequence of length N, its *N* − *k* + 1 *k*-mers were embedded into vectors of length d. With these *k*-mer embeddings, we calculated a weighted average by summing across all *k*-mer embeddings (element-wise), down-weighting high-frequency *k*-mers, and dividing by the total number of *k*-mers (*N* − *k* + 1). The sequence embedding was then obtained be subtracting its projection to its first principal component (denoising).

#### Taxa separate in the sequence embedding space at phylum and genus levels

**Fig 1:**
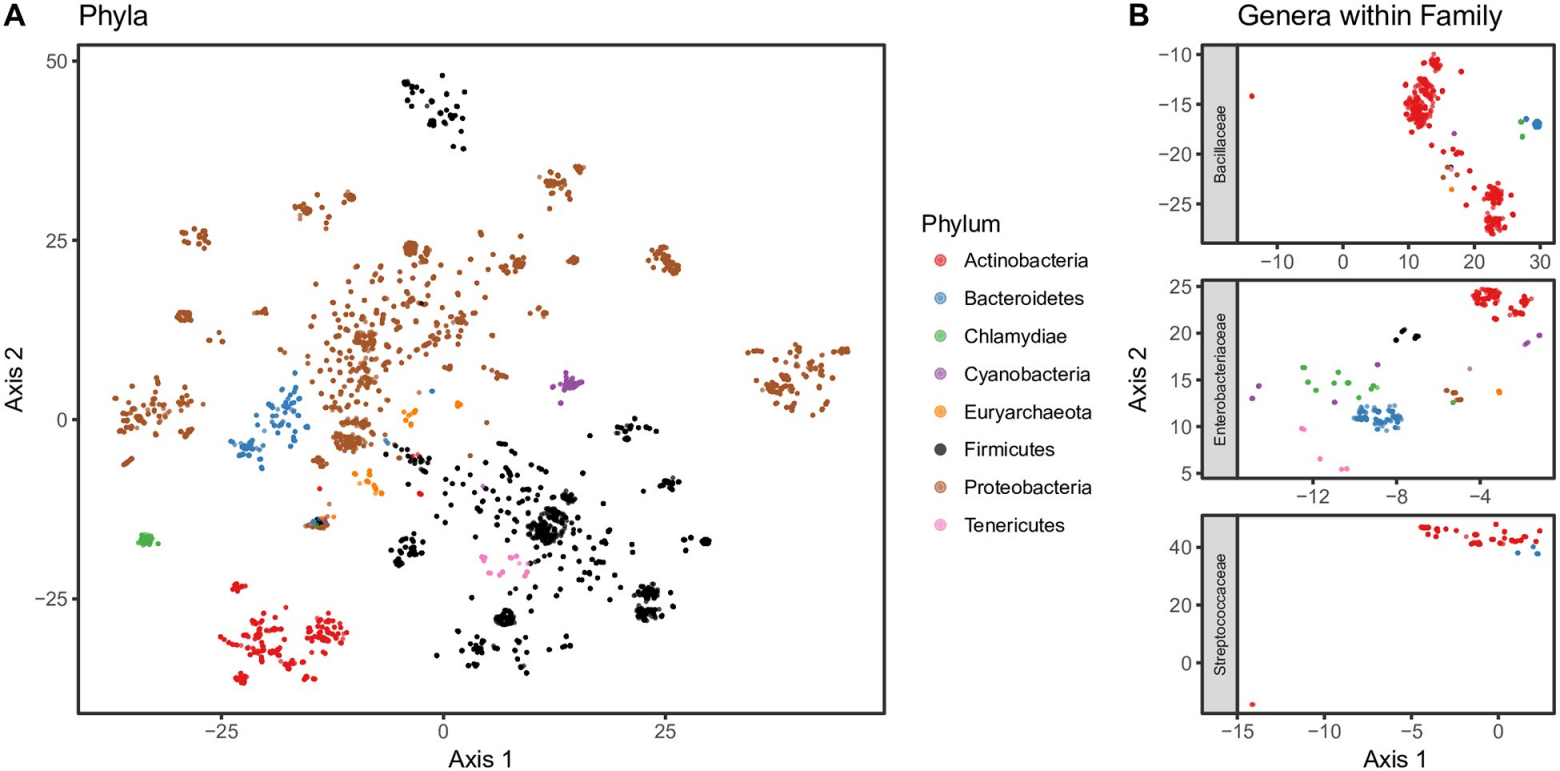
t-SNE projection of sequence embeddings from KEGG 16S sequences. Embedding results were generated using 256 dimensional embeddings of 10-mers that were denoised. A: 2-dimensional projection via t-SNE of the sequence embedding space from 14,520 KEGG 16S sequences. The position of each sequence (points) are colored based on their phylum designation. B: t-SNE projection of sequences that belong to different genera within the same family.

To visualize the sequence embedding space, we performed dimensionality reduction with t-distributed stochastic neighbor embedding (t-SNE) [41]. Fig 1 shows the 2-dimensional projections of 256-dimensional sequence embeddings that were formed by averaging 10-mer (*k*-mer where k = 10) embeddings. Shown are the eight most abundant phyla (A). Sequences (points) grouped as a function of their phylum classification. When we focus on genus classifications within a family, grouping persists. For example, within Bacillaceae (B, top), sequences from *Geobacillus spp*. (blue) separated from *Bacillus spp*. (red); within Enterobacteriaceae (B, middle), sequences from *Yersinia spp*. (red) separated from *Klebsiella spp*.; and within Streptococcaceae (B, bottom), sequences from *Streptococcus spp*. separated from *Lactococcus spp*. These observations suggest that the sequence embedding preserves genus-level resolution.

**Fig 2:**
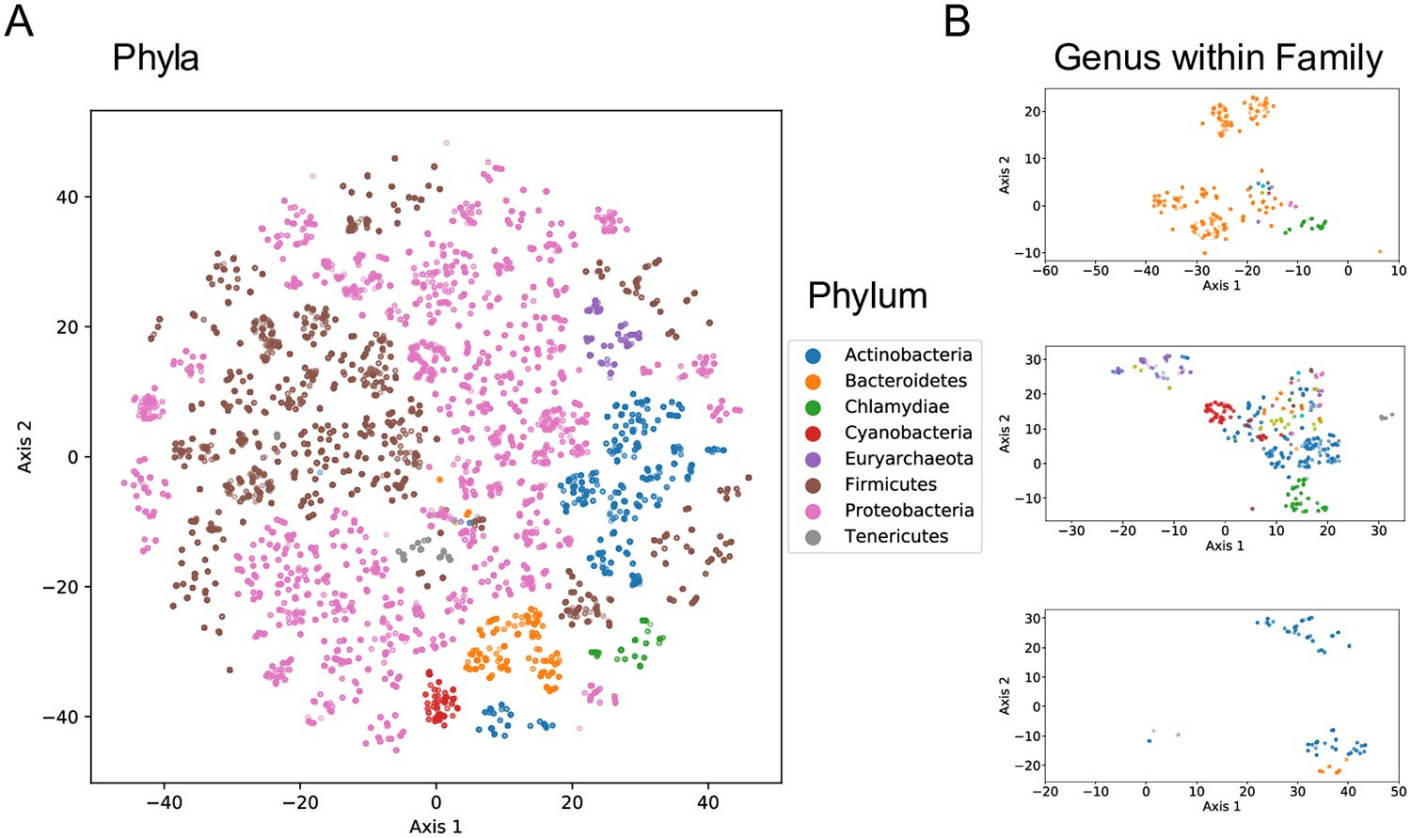
t-SNE projection of 6-mer frequency table from KEGG 16S sequences. A: 2-dimensional projection via t-SNE of the sequence embedding space from 14,520 KEGG 16S sequences. The position of each sequence (points) are colored based on their phylum designation. B: t-SNE projection of sequences that belong to different genera within the same family.

As a baseline, we visualized the t-SNE projection of sequence embeddings using *k*-mer frequencies as features (with *k* = 6) in Fig 2. We chose *k* = 6 because larger *k*, e.g., *k* = 10 resuls in a frequency table too large and hence too computational expensive for t-SNE to complete in a reasonable time. As shown in Fig 2, the k-mer method can separate the sequences from different phyla. Also, the projections using *k*-mer features and embedding features are similar in terms of cluster quality which indicts that embedding method can provide a lower space representation without much information loss.

#### Pairwise cosine similarity between sequence embeddings agrees with pairwise sequence alignment identity

**Fig 3:**
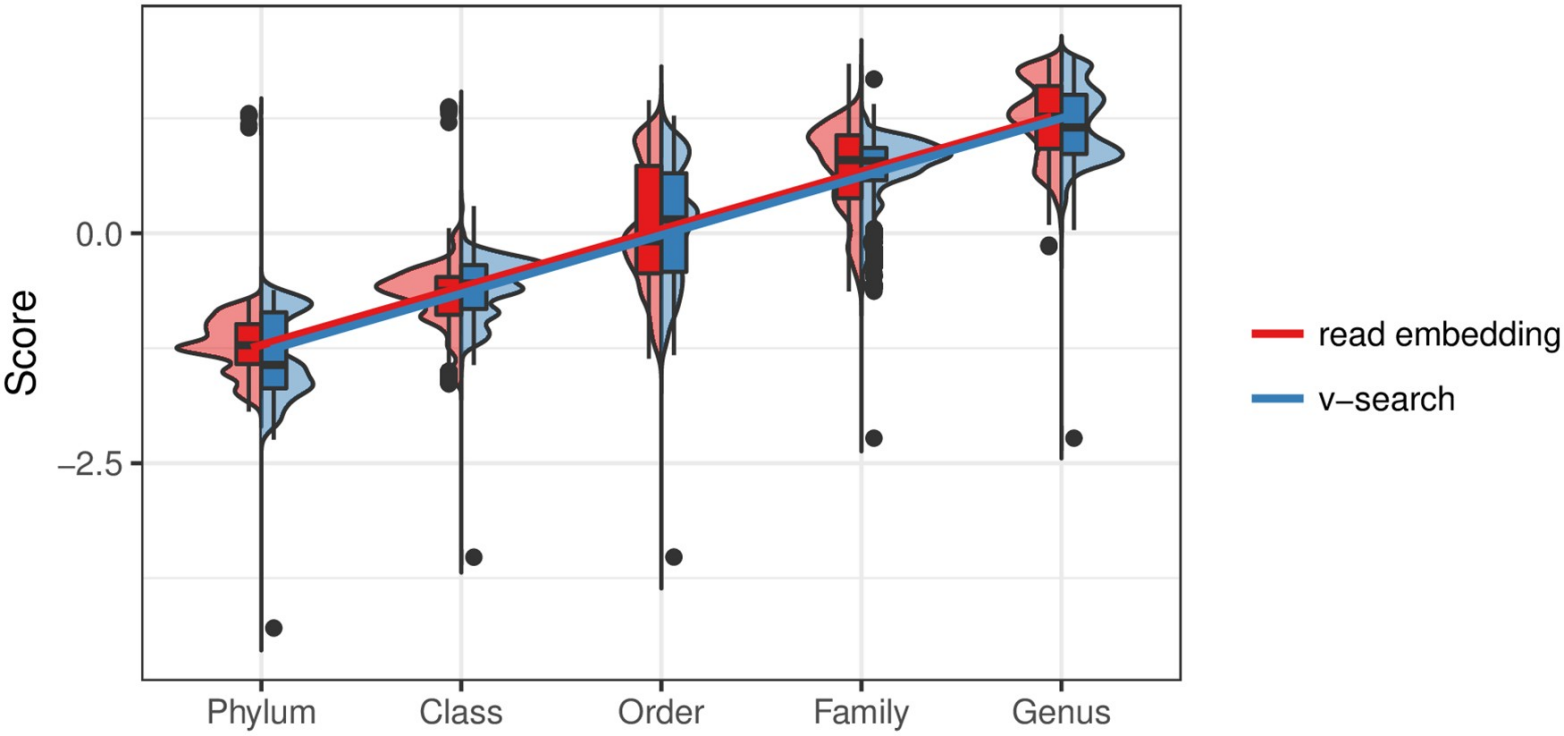
Within-taxon distribution of pairwise sequence alignment similarity verses pairwise embedding similarity. Embedding results were generated using 256 dimensional embeddings of 10-mers that were denoised. For a given taxonomic level, the red violin plots represent the distribution of pairwise cosine similarity between all sequence embeddings from the 14,520 KEGG 16S rRNA sequences, whereas the blue violin plots represent the distribution of pairwise nucleotide sequence identity using global alignment via VSEARCH. Both sets of scores were z-scored to make them visually comparable. Linear regression best fit lines are shown to ease interpretation.

With the 2,962 sequence embeddings shown as points in Fig 1B, we obtained their nucleotide sequences and performed pairwise global sequence alignment via VSEARCH [42]. These sequences belonged to Bacillaceae, Streptococcaceae, and Enterobacteriaceae families. Our objectives were to (1) determine, for a pair of sequences, if the distance (cosine similarity) between their sequence embeddings was equivalent to the distance (alignment score) between their nucleotide sequences and (2) quantify the variation in which distances between sequence embeddings vary as a function of taxon. We expected that because sequences are more similar among closely related taxa (such as taxa found within the same genus), the average pairwise cosine similarity between sequences in a lower taxonomic level should be larger. Fig 3 shows the (z-scored) within-taxon distributions of (1) pairwise cosine similarity between sequence embeddings and (2) pairwise percentage of identity between nucleotide sequences using VSEARCH. The results suggest that sequence embeddings behave similarly to sequence alignment in terms of pairwise distance, and as we move down the taxonomic hierarchy, the distance z-scores increased by 0.629 (*p* < 0.001, *R*^2^ = 0.785) and 0.636 (*p* < 0.001, *R*^2^ = 0.803) standard deviations for sequence embeddings and VSEARCH, respectively. Moreover, within genus, the standard deviation of scores for either approach (genus sequence embedding sd = 0.415; genus VSEARCH alignment sd = 0.399) is similar, suggesting the spread of distances between sequences from the same genus is equivalent between approaches. Therefore, this evidence shows that sequence embeddings are similar to global alignment at distinguishing intra-genus differences among sequences. We performed the same simulation using the frequencies of 6-mers as features (4^6^ total features). The results were similar: *p* < 0.001, *R*^2^ = 0.775. Note that we were unable to perform the same comparison using 10-mers due to the computational demand in terms of memory.

#### Embedding clusters of sequences is comparable to calculating their consensus sequence

**Fig 4:**
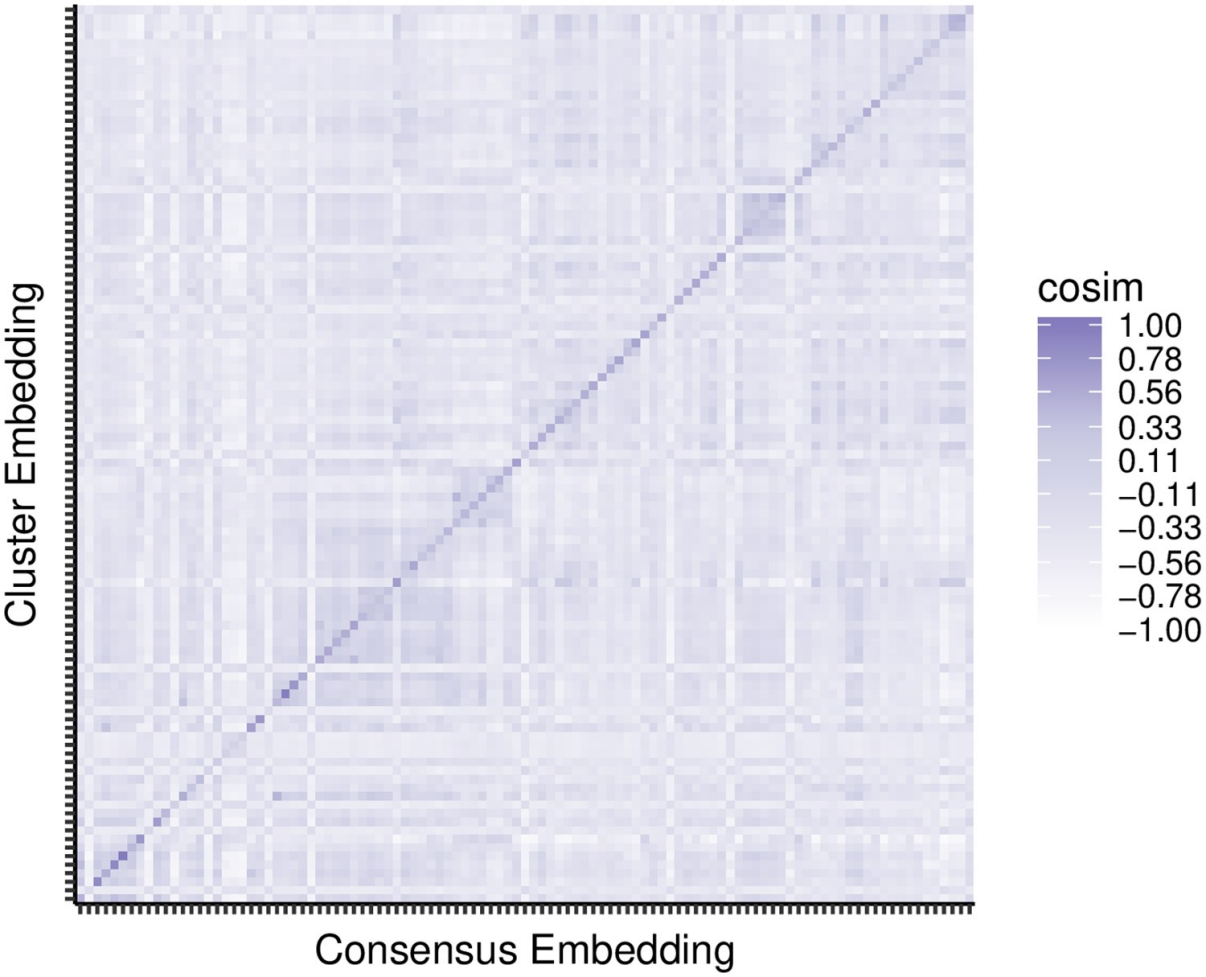
Agreement between consensus sequence embeddings and their cluster embeddings. For each cluster, all KEGG 16S sequences were embedded into a cluster embedding, and the cluster’s VSEARCH consensus sequence was embedded into a consensus embedding. The pairwise cosine similarities between all consensus and cluster embeddings are shown. They are sorted based on the (arbitrary) index for cluster membership. Darker shading indicates larger cosine similarity betweens cluster and consensus embeddings. Thus, the dark diagonal represents that the cluster embedding is similar to the consensus embedding for that cluster.

Given that the sequence embeddings behaved analogously with alignment, we hypothesized that a sequence embedding was equivalent to performing global sequence alignment and calculating a consensus *k*-mer sequence (the majority nucleotide in each column of a global alignment). By extension, averaging together multiple sequence embeddings could be thought of as calculating a consensus sequence of their nucleotide sequences. To verify this, we used VSEARCH to cluster the 14,520 KEGG 16S sequences and then obtained the consensus sequence from each of the resulting 176 clusters. We calculated (1) the sequence embedding for each consensus sequence and (2) the cluster embedding for all sequences within a cluster (*i.e*., a weighted mean sequence embedding for a given cluster, but the projection to the first principal component removed only after the complete cluster embedding is constructed). We expected that if an embedding of multiple sequences was in fact similar to calculating the consensus sequence of their nucleotide sequences, then the cosine similarity would be largest between the sequence embedding of a cluster’s consensus sequence and its cluster embedding. The cosine similarities between the consensus sequence embeddings and each cluster embeddings are shown in Fig 4. The largest cosine similarity occurs on the diagonal, between consensus and cluster embeddings pertaining to the same cluster. This observation suggests that an embedding of multiple sequences can be viewed as a consensus sequence.

#### Clustering the embeddings yield higher fidelity clusters for species than for higher taxonomic levels

To better understand how well the embedding space represented sequence data, particularly in its ability to resolve subtle differences among sequences in terms of some similarity statistics, we clustered the KEGG 16S sequence embeddings. We used *K*-means [43], which iteratively clusters sequence embeddings into the closest centroid and updates the centroid based on each cluster it contains. The free parameter K was chosen according to the total number of taxa in a given taxonomic level. For example, at the species level, there were 385 unique taxa; thus, we set K to 385, which resulted in 385 *K*-means generated clusters.

Table 1 shows the clustering performance for one of the embedding models (10-mers, 256 dimensions, denoised). *K*-means performed best at the species level for all metrics. For higher taxonomic levels, performance declined. This shows that the sequence embedding preserved short- and medium-term distance better than long-term distance—that is, similar sequences remain close in the embedding space. While the similarity between sequences belonging to different phyla is not well preserved in the embedding space, the clustering performance still suggests that the sequence embeddings are informative and capable of preserving the sequence similarity among nucleotide sequences.

**Table 1:** Clustering analysis of KEGG sequence embeddings.

Taxon	K	P	C	O	F	G	S
# of Taxa	2	35	77	143	281	697	385
K	2	35	77	143	281	697	385
Homogeneity	0.11	0.88	0.94	0.94	0.96	0.97	0.98
Completeness	0.02	0.42	0.56	0.78	0.79	0.83	0.94
ARI	-0.01	0.24	0.32	0.36	0.43	0.47	0.84
AMI	0.02	0.42	0.55	0.66	0.75	0.76	0.91
NMI	0.04	0.61	0.72	0.80	0.87	0.90	0.96

We also applied the same clustering analysis on 6-mer frequency table (Table B in S1 Appendix). We can see that our proposed embeddings outperform 6-mer frequency table significantly in terms of Adjusted Rand index (ARI). This indicates that by leveraging sequence context/neighborhood information, we can generate more meaningful clusters using our proposed embedding.

### Evaluation of sequence embeddings on 16S rRNA amplicon sequences from the American Gut project

**Fig 5:**
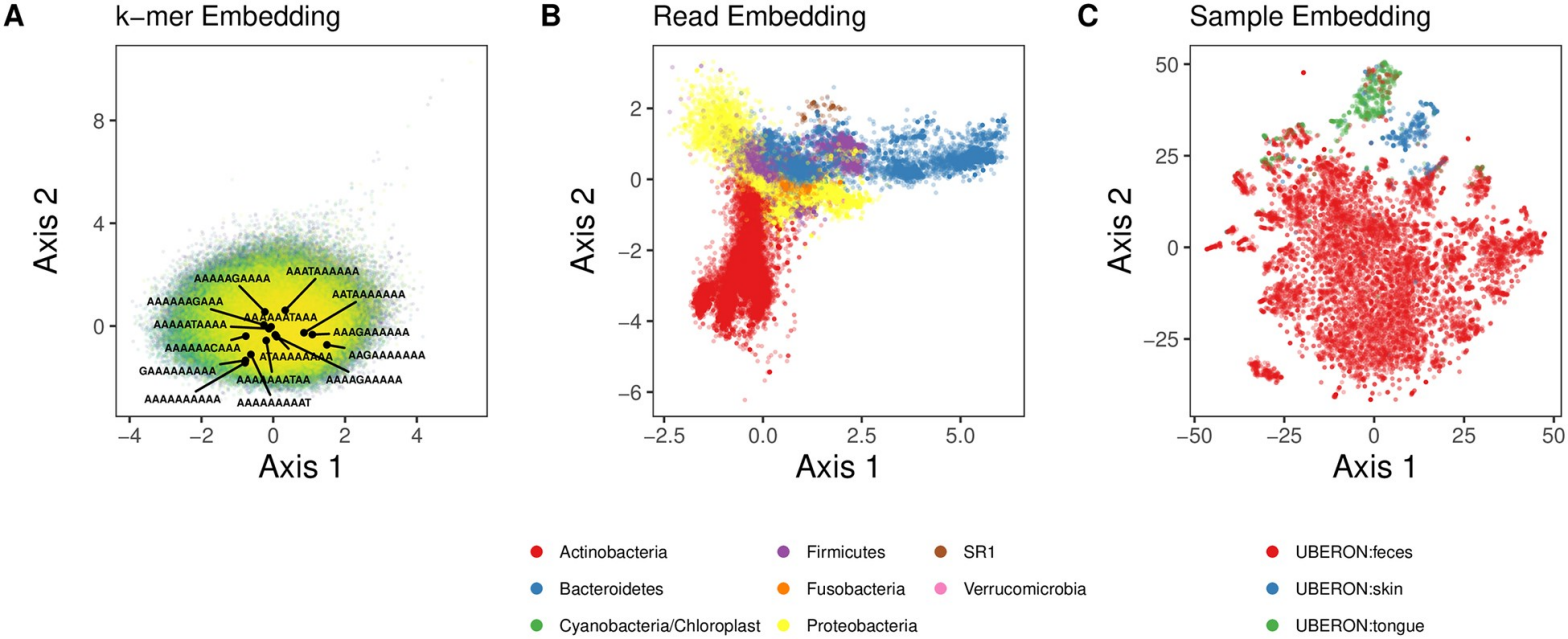
Lower dimensional projections of *k*-mer, sequence, and sample embeddings. Embedding results were generated using 256 dimensional embeddings of 10-mers that were denoised. A: A 2-dimensional projection via independent component analysis of the 10-mer embedding space from the GreenGenes training sequences. 406,922 unique 10-mers are shown. The position of 10-mers that differ by one nucleotide from AAAAAAAAAA are labeled to demonstrate that it is not simply sequence similarity that is preserved, since these sequences span a wide range in the embedding space. The *k*-mers were sorted alphabetically and ranked; the alphabetical progression of the indexes are shaded from yellow to green. B: A 2-dimensional projection via independent component analysis. 705,598 total sequences embeddings from 21 randomly chosen American Gut samples (7 from each class) are shown. The position of each sequence (points) is colored based on its phylum designation (only the 7 most abundant phyla are shown). C: 2-dimensional t-SNE projection of the 11,341 American Gut sample embeddings. The position of each sample (points) is colored based on its body site label.

Next we aimed to try our embedding approach on empirical data. We obtained sequencing data of microbiota from three body sites sequenced by the American Gut project [18]. After preprocessing, 11,341 samples from each of three body sites (fecal, skin, oral) were embedded. Unlike the KEGG 16S sequences described above, the fact that these are reads (and not full-length 16S rRNA sequences) presents a new challenge in that they are significantly shorter, spanning only 125 nucleotides per read; thus, each read is composed of, at most, 116 *k*-mers for a 10-mer embedding. The *k*-mer, sequence, and sample embedding spaces are shown in Fig 5. There was clear grouping among samples from body sites (Fig 5C) and phyla for sample and sequence embeddings, respectively (Fig 5B). For the *k*-mer embedding, discerning any meaningful patterns is more difficult since the embedding simply encodes contextual information for each *k*-mer. We were interested in the relative position of single nucleotide differences for a given *k*-mer. In Fig 5A, we mark the position of every 10-mer present that differs from AAAAAAAAAA by one nucleotide. As expected, despite subtle differences among the 10-mers, their relative position in the embedding space is broad, suggesting that each 10-mer’s context significantly influenced its embedding.

**Fig 6:**
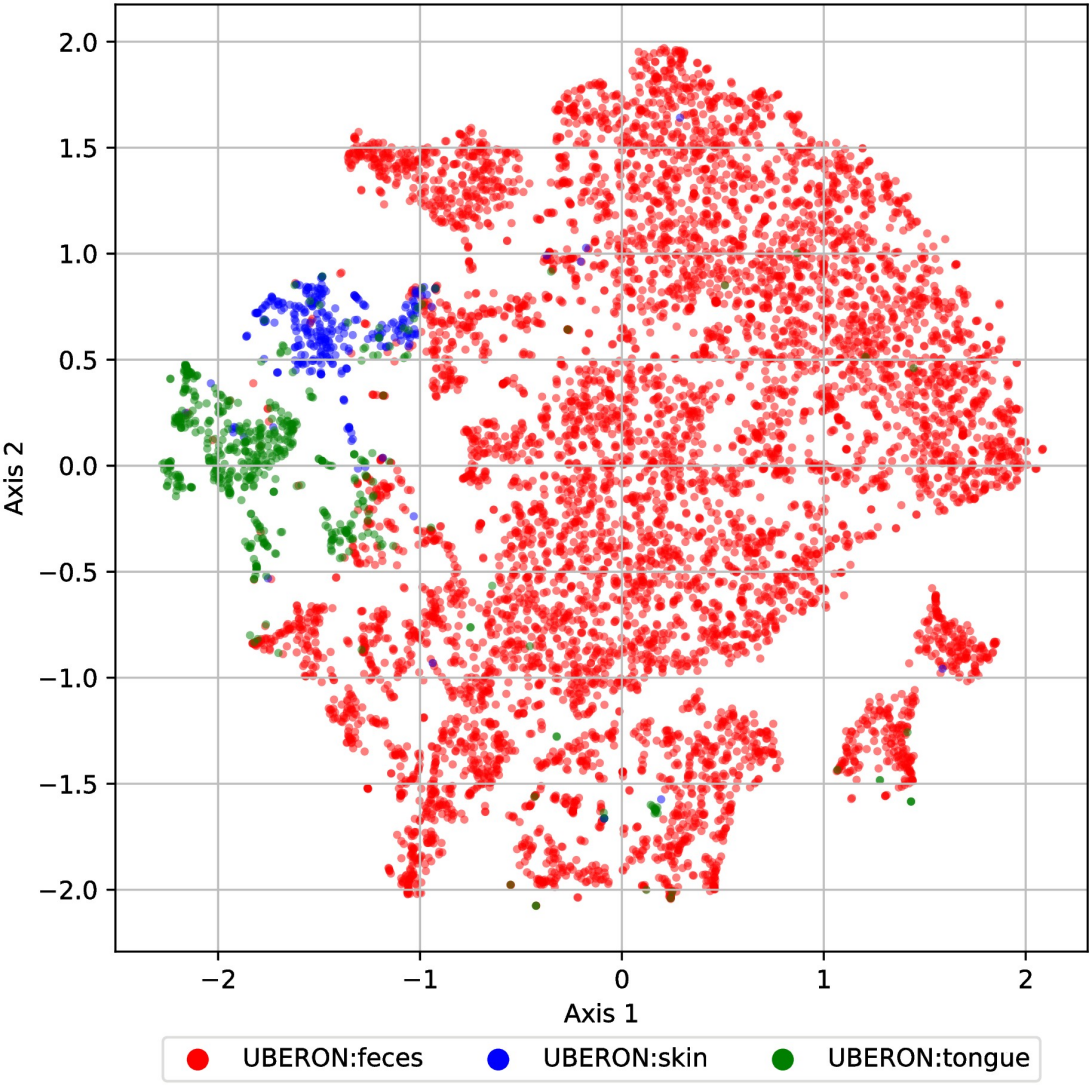
Lower dimensional projections of 6-mer sample embeddings using k-mer method. 2-dimensional t-SNE projection of the 11,341 American Gut sample embeddings based on k-mer method (where *k* = 6). The position of each sample (points) is colored based on its body site label.

As a baseline, we visualized the t-SNE projection of each sample’s vector of *k*-mer frequencies (with *k* = 6) (Fig 6). We choose *k* = 6 based on classification results shown in Table 2, which indicated that the best performing *k* using *k*-mer frequencies was *k* = 6. Note that 10-mers were too computational expensive for t-SNE. Fig 6 shows that using *k*-mer features, one can separate body sites fairly well. There is no substantial difference compared with Fig 5, which indicts that the embedding method does not result in much information loss.

**Table 2:** Sample embedding classification performance.

Features	Acc	Prec	Rec	F1
OTUs	0.922	0.958	0.891	0.922
Genera	0.934	0.960	0.905	0.931
Families	**0.938**	**0.963**	**0.916**	**0.939**
Orders	0.916	0.899	0.881	0.890
4-mers	0.967	0.917	0.956	0.935
6-mers	**0.979**	**0.942**	**0.969**	**0.953**
8-mers	0.964	0.916	0.945	0.929
Embeddings (4-mers, raw)	0.967	0.916	0.956	0.933
Embeddings (4-mers, denoised)	**0.972**	0.910	**0.963**	0.933
Embeddings (6-mers, raw)	0.958	0.900	0.943	0.920
Embeddings (6-mers, denoised)	0.945	0.905	0.925	0.914
Embeddings (10-mers, raw)	0.964	0.909	0.945	0.926
Embeddings (10-mers, denoised)	0.960	0.929	0.945	0.936
Embeddings (15-mers, raw)	0.968	**0.937**	0.957	**0.946**
Embeddings (15-mers, denoised)	0.968	**0.937**	0.957	**0.946**
Pseudo-OTUs (10-mers, denoised)	**0.977**	**0.971**	**0.964**	**0.968**

#### Sample embeddings show negligible performance loss compared to Operational Taxonomic Unit (OTU) abundances for classification

We evaluated testing performance using 256-dimensional sample embeddings as features to classify body site (fecal, skin, oral). We used a multinomial lasso classifier [44, 45] to obtain a sparse set of regression coefficients that we could use to interrogate predictive nodes at both sequence and *k*-mer embedding levels. We compared generalizability (testing performance) of different sets of features: (1) QIIME-generated [11] OTU abundances (centered-log-ratio (clr) transformed [46, 47]), (2) OTU abundances collapsed into various taxonomic levels (genus, family, order), (3) k-mer frequencies (4-mers, 6-mers, 8-mers), (4) 1000 pseudo-OTUs (clr transformed) that were generated by clustering sequence embeddings, and (5) 256-dimensional sample embeddings generated with 4-, 6-, 10, and 15-mers (Table 2). The rationale behind generating pseudo-OTUs was to more explicitly represent sequence abundances compared to averaging sequence embedding vectors, which yields a consensus sequence embedding that is weighted as a function of sequence frequency. All feature sets performed well, with the pseudo-OTUs performed best (F1 = 0.968) followed by the 6-mer frequency table 0.953) and then the embeddings using 15-mers (0.946 for both raw and denoised embeddings). The dimensionality of the k-mer feature space scaled exponentially (4^k^), which limited our ability to perform lasso cross validation for *k* > 8 due to memory demands. Using taxonomic relative abundance features yielded the worst performance in terms of balanced accuracy among any feature set (Acc = 0.916 → 0.938), and collapsing OTUs into order (110 unique features) yielded the worst performance in terms of F1 sore. Of the taxonomic abundances, family level abundances performed best (Acc = 0.938, F1 = 0.939), which had a comparably sized feature space (247) to the embedding space (256).

By using OTUs, the number of features used to classify a sample is 21,749 (generated via QIIME). The dimensions reduce even further with genera, family, and order taxonomic levels which correspond to 775, 247, and 110 number of features respectively. Our neural network takes 4-, 6-, or 10-mers as input and produces d-length vector representations of the sample as an intermediary output before the final classification of sample type. Assuming *d* = 256 (based on our benching results), using these 256-length embedded vectors, we are only using slightly more features than the 247 family feature vector for classification and significantly fewer than the over 21,000 OTU feature vector. In addition, we created 1000 pseudo-OTUs by clustering 256-length sequence-embedded vectors. Every time an embedded vector matched to one of the 1000 OTUs, its count was incremented to create a 1000 pseudo-OTU count table. The pseudo-OTUs (derived from the embedded vectors) yielded excellent classification with only 1000 features (a little more than genus-level OTUs but much fewer features than the full OTU table). In fact, all methods obtained better accuracy than just using OTUs with the embedded vectors using less features.

Overall, these results suggest that generating sample embeddings yields predictive features that remain generalizable despite drastically reducing the dimensionality of the data. Without dimensionality reduction, *k*-mer frequencies are oftentimes too memory-intensive, especially when *k* is large. This hurts interpretability since we cannot link genomic structure (using long spans of nucleotide sequences) to sample level or taxonomic information. Also, embeddings provide a dimensionality reduction approach that performs at the very least comparably (depending on the parameterization) to taxonomic abundances, but does so using a feature space that is more interpretable, capturing both nucleotide sequence information and *k*-mer context.

#### Specific sequences activate specific nodes and these activations are a function of the sequence’s taxon and the sample’s body site

We next aimed to determine (1) how the sample embedding matured as sequence embeddings from different taxa were introduced and (2) how specific dimensions (nodes) in the embedding space (the hidden layer) influenced the final sample embedding. When simply optimizing for classification performance, it is not unreasonable to treat a neural network, or any machine learning model, as a black box, but with a better understanding of what these models are learning, the potential for novel applications increases. Hence, the interpretation of the hidden layers in neural networks is an active area of research [35, 48–51]. One approach is to train a model and then traceback from its prediction to identify key features influential in the classification decision [48, 52]. By performing classification with the sample embedding treated as features, we can traceback to disentangle how key nodes represented the embedding (*e.g*., whether particular nodes activated as a function of taxon), as well as discern the impact specific reads, and hence taxa, had on both the sample embedding and classification.

We obtained the same sparse set of regression coefficients that were estimated via lasso to classify body site using sample embeddings of the American Gut data (see above). We calculated read activations (the linear combination of the lasso regression coefficients and the sequence embeddings) and then inspected how the cumulative sum of these activations affected the final body site classification (fecal, tongue, or skin).

**Fig 7:**
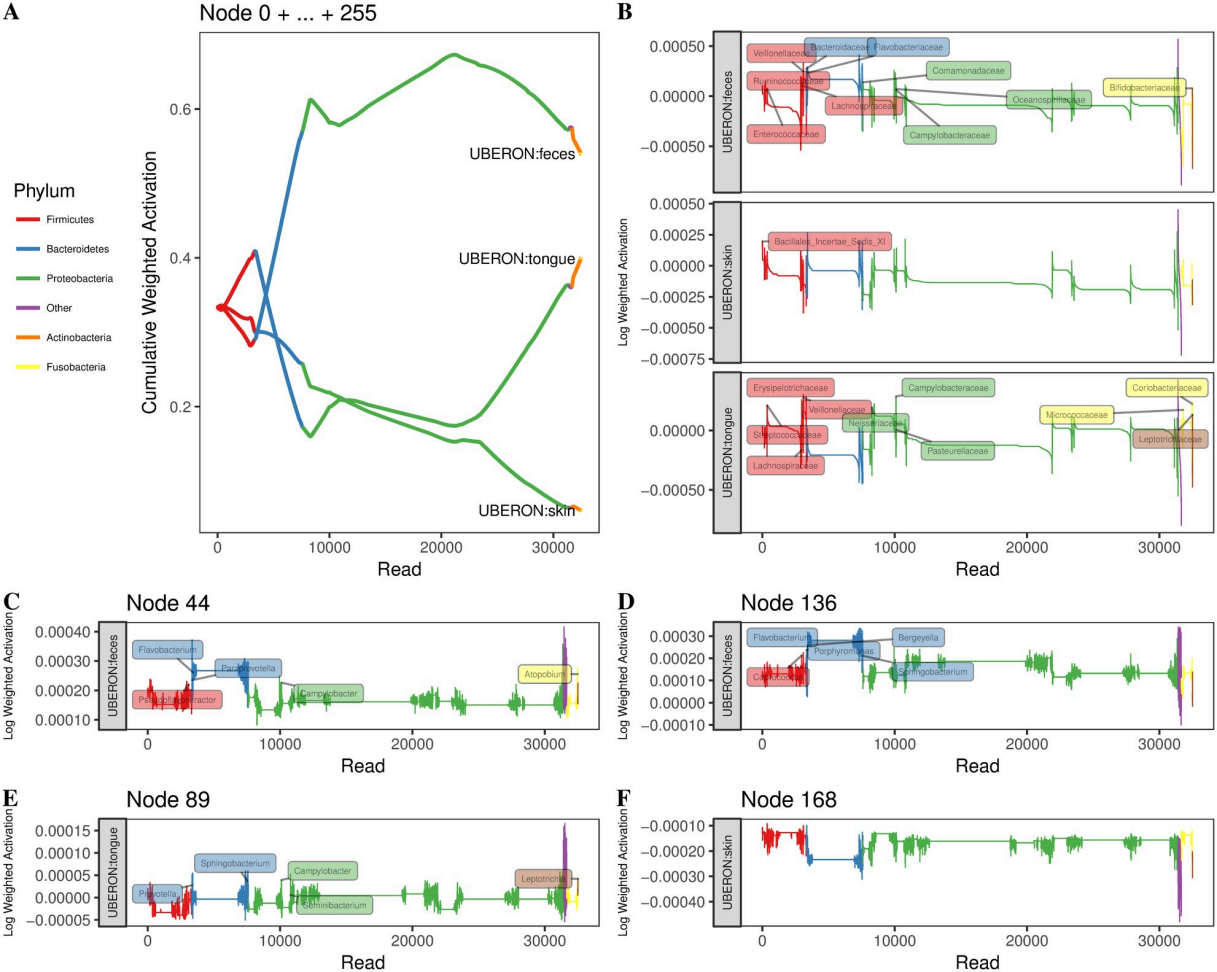
Maturation of the body site classification decision as sequence embeddings are introduced. Embedding results were generated using 256 dimensional embeddings of 10-mers without denoising (since the number of reads varies throughout the figure). One American Gut tongue sample is shown, which was misclassified by lasso as “fecal.” Read activations are defined as the linear combination of a given sequence embedding and the regression coefficients obtained from lasso for a particular body site. A: The trajectory of the body site classification decision via multinomial lasso. The cumulative activation is the sum of all read activations across all nodes (dimensions of the embedding) up until the introduction of a specific read. A body site is favored when it has the largest cumulative activation of the three body sites. Reads were sorted and introduced based on their taxon (phylum designations are color coded). B: The (non-cumulative) activations (across all nodes) for body site as reads are introduced (with no accumulation from previous reads). Genera labels are shown for reads with large activations. C-F: The (non-cumulative) activations for individual nodes and specific body sites as reads are introduced.

Fig 7 shows the trajectory of the classification decision for a single tongue sample that was misclassified as fecal. The sequences were sorted and added to the embedding in order, based on their taxonomic classification (independently obtained via the naive Bayes RDP classifier). Fig 7A and 7B) show the trajectory of all nodes (their sum) in the embedding, and thus represents the overall classification decision as reads are introduced. Fig 7C–7F show the activations for individual nodes that greatly impacted the classification decision (*i.e*., they had large activations). Note that only Fig 7A shows the cumulative trajectory; Fig 7B–7F show the activations specific for a given read (index).

The initial set of reads introduced to the sample embedding were 3327 reads belonging to the phylum Firmicutes. This set of reads drove the classifier to favor a “skin” classification. Highly influential genera among these reads include *Granulicatella*, *Gemella*, and *Streptococcus*, which is consistent with work linking these genera to microbiota inhabiting the nares [53], as well as work demonstrating shifts in *Gemella* and *Streptococcus* occurring in response to atopic dermatitis [54]. As 4263 Bacteroidetes reads were introduced to the sample embedding, the classifier began to favor a “feces” classification. However, influential reads predominantly belonged to Flavobacteriaceae, a family often associated with oral microbiota [55, 56]. Perhaps during training, the encoding of this family became associated with k-mers belonging to gut sequences. Thus, the fact that the classifier incorrectly associated these oral microbiota with the fecal body site likely influenced the classifier to classify the sample incorrectly. The introduction of Proteobacteria influenced the classifier differently at different stages, depending on the type of Proteobacteria introduced. Initially, reads assigned to the family Comamonadaceae (x-axis read index = 7591), a bacterial family associated with gut microbiota [57], reinforced the fecal classification. Favorability towards tongue increased upon introduction of reads (read index = 8641) belonging to the genera *Neisseria*, and to a lesser extent, *Campylobacter* and *Haemophilus*, all of which have been linked to oral microbiota [55, 56]. *Acinetobacter* reads (read index = 10866) that were favorable to a fecal classification were then introduced. *Acinetobacter* has been linked dental plaque accumulation, nosocomial respiratory disease, poor cholesterol profiles, gut dysbiosis, and colorectal cancer [58–61]; thus, *Acinetobacter*’s association with both oral and gut microbiota may have also contributed to the misclassification. Finally, *Pseudomonas* and *Stenotrophomonas* reads (read index = 21189), as well as a short-lived contribution of *Atopobium*, *Rothia*, and *Actinomyces* reads from the phylum Actinobacteria (read index = 31655) shifted the classifier closer to a tongue classification, which is consistent with literature [5, 56]. However, these oral shifts were unable to outweigh the misclassifications from the Flavobacteriaceae, and thus “fecal” was the final classification decision.

When we focused on individual nodes (dimensions in the embedding space), we were able to link nodes to specific taxa. For example, nodes 44 and 136 received non-zero regression (lasso) coefficients for classifying samples as fecal. These nodes had large activations for sequences stemming from *Prevotella* and *Flavobacterium* from the Bacteroidetes phylum, but node 136 was specific for large Bacteroides activations (sequence index = 3328-3336). Node 168, on the other hand, was important in skin classifications (non-zero regression coefficients) and had relatively large activations for sequences from Firmicutes phyla, specifically *Staphylococcus*, *Gamella*, *Oribacterium*, *Veillonella*, and *Granulicatella*.

#### *k*-mers associated with Lachnospiraceae genera were specific for different body sites and specific regions of the 16S rRNA gene

Because the embedding was trained with *k*-mers, and each sequence or sample embedding therefore amounts to a weighted sum of *k*-mer embedding vectors, we found it necessary to traceback further to the encoded *k*-mer information. This is similar to the question found in natural language processing where feature interpretation is necessary to elucidate how the neural network represents saliency and compositionality [35]. Trying to extract meaning from sequences of nucleotides is obviously a different question, but nevertheless, much can be learned from natural language processing in understanding the context of a given *k*-mer, its co-occurrence profile, and how sequential sets of *k*-mers influence a classification decision differently than their constituent parts. For example, recent work has attempted to identify regulatory regions in genomic sequences in this manner [49].

Thus, our objectives were to identify (1) *k*-mers that strongly influence the performance during sample-level (body site) classification and (2) characteristics of the sequences that contain these *k*-mers, such as the sequence’s taxonomic classification vis-á-vis the neighborhood in which important *k*-mers occur. We again obtained the sparse set of regression coefficients that were estimated via lasso to classify body site using sample embeddings of the American Gut data. We calculated *k*-mer activations (the linear combination of the lasso regression coefficients and the *k*-mer embeddings) and then selected the top-1000 *k*-mers with largest activations for classifying skin, tongue, or fecal body sites. These *k*-mers, when present in a sequence, have the single greatest influence on the classification outcome for a given sample, although it should be noted that the final classification decision is predominantly driven by high-frequency (after weighting) *k*-mers with relatively large activations.

Here we focus on the classification of skin samples. For each of the 3 sets (skin, tongue, fecal) of 1000 *k*-mers with large activations, we identified which reads from American Gut skin samples contained these *k*-mers. From the 11,838,849 reads in the 282 skin samples, 241,006 reads contained any of the top-1000 *k*-mers with large activations for skin, whereas 74,240 and 197,505 reads contained any of the top-1000 *k*-mers with large activations for tongue and fecal classification, respectively. Only 2781 reads contained *k*-mers from both skin and fecal-associated *k*-mers (the intersection), whereas 5056 reads contained both skin and tongue-associated *k*-mers. Also, 626 of the 2781 reads with both skin and fecal-associated *k*-mers belonged to only two samples (221, 405). Interestingly, the sample with 221 reads that contained skin- and fecal-associated *k*-mers was misclassified by the lasso classifier (using the parameterization optimized during training), but any general trend between misclassification and the proportion of high-activation *k*-mers was not apparent. This finding is consistent with how the embedding activations affect classification, as we demonstrated above; *k*-mer activations incrementally influence the classifier, with no single *k*-mer embedding having substantial impact on the ultimate classification decision. Still, as we have shown, the *k*-mer profiles associated with particular body sites are distinct.

**Fig 8:**
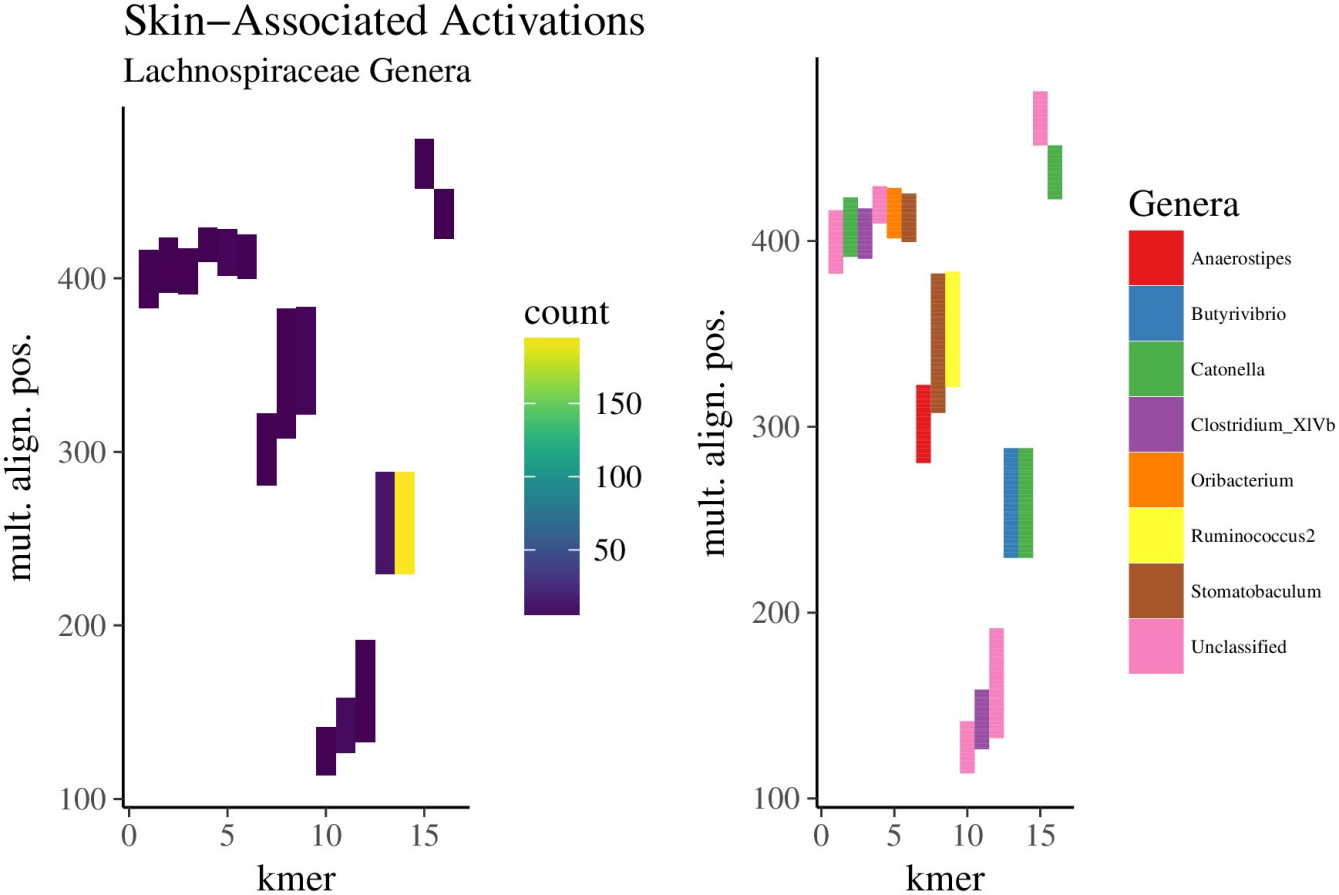
Regions within Lachnospiraceae reads among in which skin-associated *k*-mers mapped. The top-1000 *k*-mers with the largest activations (the linear combination of the *k*-mer embedding and regression coefficients obtained via lasso) for skin were identified. A random sample of 25,000 reads (from skin samples) containing these *k*-mers underwent multiple alignment. Shown is the relative position of *k*-mers found only in Lachnospiraceae reads from skin samples. The position of a given *k*-mer in the alignment spans its entire starting and ending position, including gaps. The frequency in which these *k*-mers mapped to positions (left column) in the multiple alignment were quantified (y-axis). The order of the *k*-mers in the heatmap (x-axis) was obtained via hierarchical clustering (Ward’s method) on Bray-Curtis distances. The Lachnospiraceae genus that most frequently occurred at a given alignment position for a given *k*-mer is colored (right column).

To further characterize the *k*-mer profiles, we associated high-activation *k*-mers with the neighborhood of their read from which they originated. For each set of reads that contained the high-activation *k*-mers, we randomly sampled 25,000 reads and performed multiple alignment. Fig 8 shows the mapping regions in the multiple alignment for *k*-mers with large activations. We only show genera belonging to the family Lachnospiraceae because focusing on genera that share a family, for example, may present interesting patterns as to how specific *k*-mers, associated with specific taxa, favor specific regions in the 16S rRNA gene. The cluster of 6 *k*-mers (index 1-6) (TTTCGGAACT, CCAGAACTGA, TTTGGAGCTA, ACTTATAAAC, AACTGTTGCT, GAAACCGTGC) that span nucleotide 500 in the multiple alignment mapped to reads from *Catonella* (120 reads), *Clostridium* (59), *Oribacterium* (27), *Stomatobaculum* (101), and two unclassified genera (340). For each of these 6 *k*-mers, we calculated their nearest *k*-mer neighbors—that is, all *k*-mer embeddings with cosine similarity greater than 0.25 (arbitrarily chosen). TTTGGAGCTA and GAAACCGTGC shared ten neighbors, whereas TTTCGGAACT, CCAGAACTGA, and ACTTATAAAC shared one, suggesting that some context (neighboring *k*-mers) was shared among these *k*-mers. This helps confirm the positional relationship seen in Fig 8. Coupling these results with our observation that intra-family genera separate in the embedding space (Fig 1), this region of the multiple alignment may pinpoint a key location of the 16S rRNA variable region that distinguishes these taxa. While we do acknowledge this claim garners additional evidence, we do feel it is worthy of future work, particularly with datasets that can yield more trustworthy taxonomic assignments. In addition, another interesting avenue for future work may involve using full length 16S rRNA sequences to identify associations between *k*-mer and sequence embeddings to elucidate which variable regions are favored by specific taxa.

### The embedding corpora

#### Many *k*-mers in the query datasets lacked embeddings

The total number of unique 6-mers and 10-mers in the GreenGenes reference database, used for training, was 4096 and 413,329, respectively. Of the 406,922 unique 10-mers in the KEGG 16S sequences, 270,676 were present in GreenGenes and thus had embeddings (*i.e*., were present in the training set), whereas all *k*-mers were present for 6-mers. Of the 1,048,576 unique 10-mers in the American Gut reads, only 413,329 were embedded. Thus, the sample embeddings extracted meaningful features and maintained high performance despite over half of the American Gut *k*-mers not being used. This begs the question: what proportion of a given dataset is required to generate a meaningful embedding? Future work should explore (1) how sensitive the embedding space is to the degree of overlap between the of training and query vocabularies and (2) whether the requirement for sufficient overlap changes when the query data consists of longer sequences.

#### Denoising sequence and sample embedding spaces removes mean background signal, which helps resolve the pairwise similarity among embeddings

**Fig 9:**
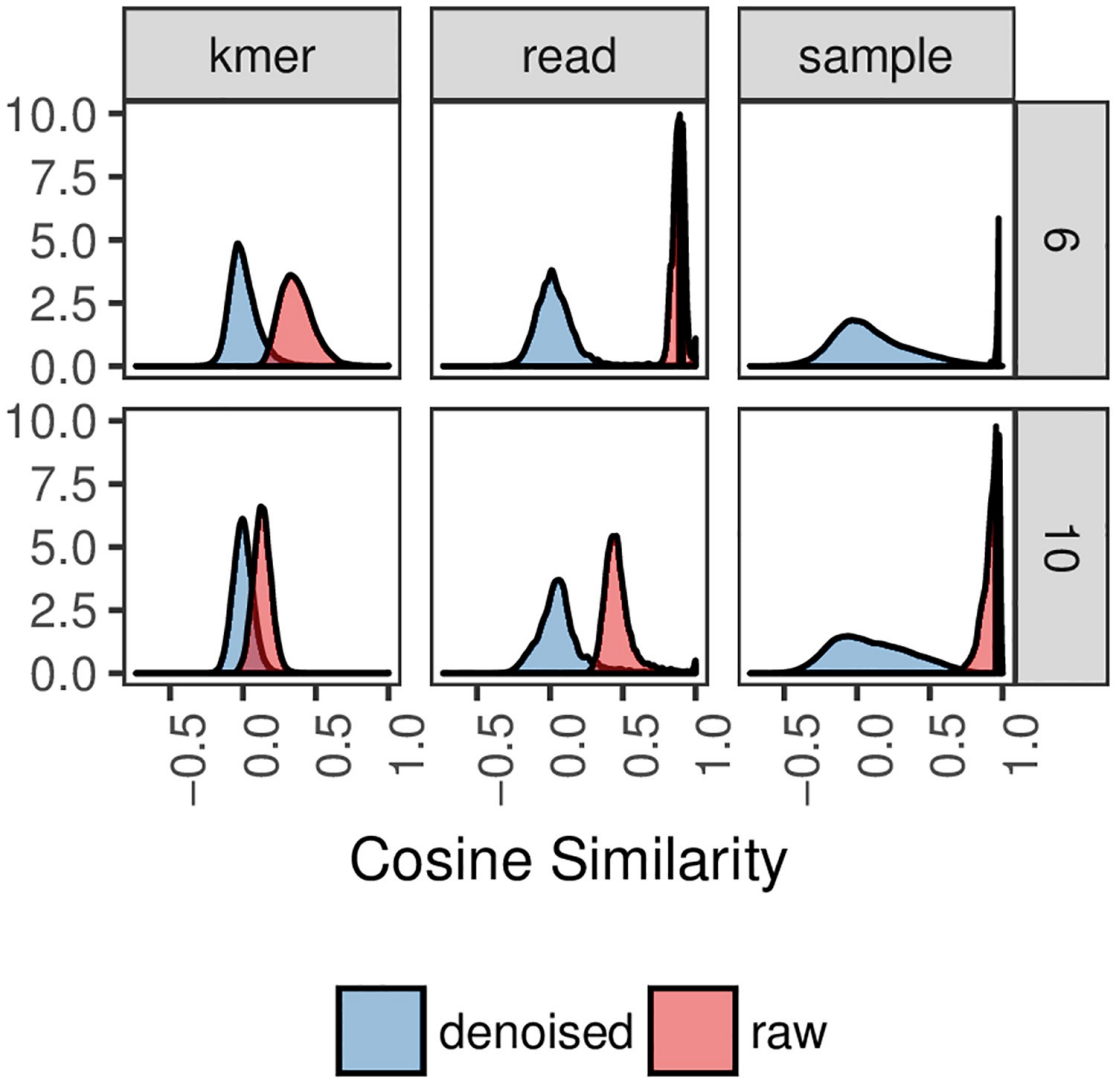
Distribution of pairwise cosine similarity for *k*-mer, sequence, and sample embeddings of the American Gut data. Sequence and sample embeddings were calculated from 21 randomly selected (7 for each body site) samples. *k*-mer embeddings were those used during training with the GreenGenes sequences. Shown are distributions of pairwise cosine similarities as a function of *k*-mer size (rows), denoising (blue versus red), and embedding space (columns).

The predominate effect of the denoising approach (removal of the projection to the first principal component) can be seen in Fig 9. Which shows the distribution of pairwise cosine similarity scores for *k*-mer, sequence, and sample embeddings using 6-mers and 10-mers. The results indicate three major trends. First, shorter *k*-mers result in larger pairwise cosine similarity between sequences, which is likely due to larger sequences being less common and, by extension, having more distinctive *k*-mer neighborhoods. Second, as more sequences are introduced to a given embedding, such as building a sample embedding compared to building a sequence embedding, the pairwise cosine similarity between sequences increases. We posit that as the number of sequences increases in an embedding, common *k*-mer embeddings begin to dominate in the same way that stop-words such as “the” may affect sentence embeddings. This, in turn, would explain the third trend: denoising mitigates this effect by removing the most common component that captures a mean background signal. Thus, we can conclude that denoising is pertinent in tasks involving cosine similarity where resolving subtle differences between sequences is paramount. Moreover, as the number of sequences introduced increases, denoising becomes more important.

### Conclusion

Here we have applied word and sentence embedding approaches to generate *k*-mer, sequence, and sample embeddings of 16S rRNA amplicon sequencing data. We present our results at a time when deep neural network approaches are readily being applied to genomic sequencing data [62]. Although these approaches have been utilized to a lesser extent in microbiome research, increased use is likely inevitable as sequencing data becomes more available. Thus, obtaining meaningful numeric representations of microbiome sequences that does not suffer from the curse of dimensionality and can act as input to various machine learning architectures is necessary.

Our work demonstrates that sequence and sample embeddings are dense, lower-dimensional representations that preserve relevant information about the sequencing data such as *k*-mer context, sequence taxonomy, and sample class. We have shown that these representations are meaningful by helping to classify taxa and samples and hence the embedding space can be exploited as a form of feature extraction for the exploratory phase of a given analysis. The sequence embedding space performs well compared to common approaches such as clustering and alignment, and the use of sample embeddings for classification seemingly results in little-to-no performance loss compared to the traditional approach of using OTU abundances. Because the sample embeddings are encoded from *k*-mer embeddings, its classification performance justifies further inquiry as pretrained input for more complex machine learning architectures such as deep neural networks. In addition, future work should aim at elucidating the effects of different training datasets and obtaining a better understanding of the feature representations at the *k*-mer level.

## Materials and methods

### Primer on word2vec

Word2vec represents words as continuous vectors based on the frequency of pair co-occurrence in a context window of fixed length. We can understand it as mapping individual words (*k*-mers in our application) to points in a continuous, higher-dimensional space, such that words with similar semantic meaning are closer to one another.

### Training data preprocessing and word2vec training

We obtained 1,262,986 full length 16S rRNA amplicon sequences from the GreenGenes database [63]. Full length sequences were used to ensure that our embeddings were region agnostic, in that the embedding would be trained on context windows found in any variable or conserved region throughout the 16S rRNA gene, permitting query sequences to be embedded irrespective to the region of the gene they spanned. For each sequence of length N, we generated all possible subsequences (*N* − *k* + 1) of length k (*k*-mers), In natural language processing terms, these *k*-mers were treated as words belonging to a corpus, and the set of all unique *k*-mers comprise the corpus’s vocabulary. k-mers with degenerate bases (bases other than ACGT) were removed. Four training sets were created for *k*-mer lengths of 4 (4-mers), 6 (6-mers), 8 (8-mers), 10 (10-mers), 12 (12-mers) and 15 (15-mers).

*k*-mer embeddings were trained using gensim’s Skip-Gram word2vec implementation over 5 epochs [64]. *k*-mers occurring fewer than 100 times were removed. We varied the parameters to generate 48 model parameterizations. Using 6-mers and 10-mers, we varied the dimensionality of the embedding (64, 128, 256); the threshold in which high frequency *k*-mers were down-sampled (0.0001, 0.000001); the number of negative samples (10, 20); and the width of the context window (20, 50). Other parameters were set to their default values. However, the model could not finish training on 8-mers, 12-mers, and 15-mers training sets within 7 days, whereas the model could finish training using other training sets within 5 days. Limited by our computational resources, we removed 8-mers and 12-mers from our analyses. In the future work, one could use other embedding implementations such as GLoVe [65]. In GLoVe, the model is trained on word-to-word co-occurrence matrix which is sparse and much smaller than the total number of words in the corpus. Therefore, the training iterations are much faster.

### Baseline: *k*-mer method

*k*-mer based 16S rRNA embedding for the host phenotype prediction has already been presented in [66]. In addition, *k*-mer profiles of samples have been shown to outperform OTU profiles in body site classification tasks [67]. *k*-mer frequencies are therefore a natural baseline for the embedding approach to be compared. Here, we created a *k*-mer frequency table for our experimental datasets. For the *k*-mer frequency table for American Gut dataset, each row is a sample and each column represent the frequency of a *k*-mer across all reads for that sample. For the *k*-mer frequency table for KEGG dataset, each row is a taxon and each column represent the frequency of a *k*-mer in the corresponding DNA sequence in KEGG dataset. These tables can then be processed and learned by other down-stream analysis tools.

### KEGG data acquisition

16,399 full length 16S rRNA amplicon sequences were obtained from the KEGG REST server (9/2017).

### American Gut data acquisition and preprocessing

188,484,747 16S rRNA amplicon reads from the American Gut project (ERP012803, 02/21/2017) underwent quality trimming and filtering. Sequences were trimmed at positions 10 and 135 based on visualizing the quality score of sampled sequences as a function of base position [12]. Then, sequences were truncated at positions with quality scores less than or equal to 2. Truncated sequences with total expected errors greater than 2 were removed. Some American Gut samples were contaminated by bacterial blooming during shipment. Contaminated sequences were removed using the protocol provided in the American Gut documentation (02-filter_sequences_for_blooms.md). Only sequences from fecal, hand, head, and tongue body sites were kept. Head and hand were merged into a “skin” category. Any remaining samples with fewer than 10,000 total reads were removed.

### American Gut OTU picking

Closed-reference OTU picking was performed with QIIME using SortMeRNA against GreenGenes v13.5 at 97% sequence identity [11]. Library size was normalized via clr, where the normalized vector of abundances $x_{s}^{*}$ for sample s was obtained by $x_{s}^{*}=\log[(x_{s}+1)/g_{s}]$, where *g*_*s*_ is the geometric mean for sample s,

$$\begin{array}{c} g_{s}={\left(\prod_{x\in s}x\right)}^{\frac{1}{\left|s\right|}}, \end{array}$$ (1)

and x is the unnormalized abundance of a single OTU.

### Embedding sequences, samples, body sites, and clusters

To generate a sequence embedding, the weighted embeddings of all *k*-mers m belonging to sequence r were summed and then normalized by the total number of *k*-mers M_r_ in sequence r. Each *k*-mer was weighted based on its frequency within the query set of sequences (sequences to be embedded, but not the sequences initially used for training). Note this down-weighting is distinct from the down-weighting used during training, which down-weighted *k*-mers based on their frequency in the training (GreenGenes) sequences. Thus, we have

$$\begin{array}{c} \varepsilon_{r}^{\left(raw\right)}=\frac{1}{M_{r}}\sum_{m\in r}\varepsilon_{m}\frac{a}{a+f_{m}}, \end{array}$$ (2)

where *ε*_*m*_ is the d-dimensional embedding for *k*-mer m, *f*_*m*_ is the frequency of *k*-mer m across the entire set of query sequences to be embedded, a is the parameter to control the degree in which *k*-mer m is down-weighted, and *M*_*r*_ is the total number of *k*-mers embedded into sequence r (*i.e*., the total number of *k*-mers belonging to sequence r that were also present in the training set and thus have embeddings). The resulting raw sequence embedding was then denoised by removing its projection to its first principle component,

$$\begin{array}{c} \varepsilon_{r}=\varepsilon_{r}^{\left(raw\right)}-\varepsilon_{r}^{\left(raw\right)}\nu \nu^{T}, \end{array}$$ (3)

where *ν* is the first principle component obtained via singular value decomposition.

For sample, cluster, or body site embeddings, the process was instead applied to all *k*-mers belonging to all sequences from a specific sample, all sequences from a specific cluster, or all sequences from all samples from a specific body site, respectively. Note that k-mers with degenerate bases were removed (bases other than ACGT); thus, some sequences received no embedding due to no *k*-mers intersecting with the training *k*-mer embeddings.

### Cosine similarity between embeddings

For an embedding A, its cosine similarity with respect to embedding B is defined as

$$\begin{array}{c} \text{cosim}\left(A,B\right)=\frac{\sum_{i}A_{i}B_{i}}{\sqrt{\sum_{i}A_{i}^{2}\sum_{i}B_{i}^{2}}}. \end{array}$$ (4)

### Lower dimensional projections of the embedding spaces

For visual exploratory analysis of the embedding space, American Gut sample embeddings were reduced to 2 dimensions via t-SNE. 21 samples (7 samples each from fecal, tongue, and skin) were randomly chosen to lessen the computational burden. Principal component analysis was not performed beforehand to further reduce the dimensionality of the embedding space, as typically done. This is because the embedding space is already a lower-dimensional representation of the original input feature space. Centering and scaling was also not performed. Perplexity was set to 50 and t-SNE was run for 1000 iterations. sequence embeddings of the 14,520 KEGG 16S sequences were explored in the same manner. For the American gut data, because the number of total sequences was large (11,838,849), and for 10-mer embeddings in general, t-SNE was impractical in terms of time and memory requirements. Thus, to project American Gut sequence and GreenGenes 10-mer embeddings to 2-dimensions, we performed independent component analysis.

### Consensus sequence analysis

The KEGG sequences were clustered using VSEARCH. Sequences with pairwise identity (as defined above) with its centroid below 0.8 were omitted from their respective cluster. We embedded the consensus sequence of each cluster (a consensus embedding), as well as all sequences belonging to that cluster (a cluster embedding). Then, the pairwise cosine similarities between all consensus and cluster embeddings were calculated.

### Generation of pseudo-OTUs

After generating sequence embeddings of the American Gut data, we randomly sampled approximately 1,000,000 sequence embeddings across all body sites (tongue, skin, gut) and used *K*-means [43] to cluster them into 1000 clusters (1000 pseudo-OTUs). We then obtained the centroids of these clusters. sequences from each sample were classified into the closest centroid/cluster. Finally, we quantified the number of sequences that were classified into each cluster and the abundance of each pseudo-OTU.

### Classification analysis

American Gut samples with body site (fecal, skin, tongue) labels were split into 90/10 training/testing sets containing 7526 and 835 samples, respectively. The training set was composed of 6729, 282, and 497 fecal, skin, and tongue samples, whereas the testing set consisted of 749, 31, and 54 samples, respectively. We performed multinomial classification using the lasso classifier with sample embeddings, clr-transformed OTUs, their top-256 principal components, or clr-transformed pseudo-OTUs as features. For training, we performed 10-fold cross validation to select the optimal value of the regularization parameter λ. We evaluated performance using the held-out testing set in terms of balanced accuracy, which adjusts for class imbalance by averaging the three accuracies for each individual body site:

$$\begin{array}{c} {\text{Acc}}^{*}=\frac{1}{3}\sum_{bs}p\left(y_{true}^{\left(bs\right)}=y_{pred}^{\left(bs\right)}\right), \end{array}$$ (5)

where *y*_*true*_ is the true label and *y*_*pred*_ is the predicated label for only samples from body site *bs*.

### Taxonomic assignment

Taxonomic assignment for both the KEGG and American Gut 16S rRNA amplicon sequences was conducted using the RDP naïve Bayes classifier [68] implemented in QIIME.

### Identification of important *k*-mers

We obtained the sparse set of lasso regression weights estimated when we performed body site classification using sample embedding (described above). Each body site (skin, fecal, tongue) had its own vector of regression coefficients. To obtain *k*-mer activations, we calculated the outer product between all *k*-mer activations and the regression coefficients: *α*_*k*_ = *ε*_*k*_ ⊗ *β*, where, for a corpus of M *k*-mers, *α*_*k*_ is an *M* × *J* matrix of *k*-mer activations for J body sites, *ε*_*k*_ is an *M* × *d* matrix of *k*-mer embeddings, and *β* is a *d* × *J* matrix of regression coefficients. Each column in *α*_*k*_ was ranked, and the top-1000 (arbitrary) *k*-mer activations for each body site were selected. For each set of 1000 *k*-mers, we identified which corresponding American Gut reads (from samples of a particular body site) contained these *k*-mers and randomly sampled 25,000 of *k*-mer-containing sequences from each body site (to ease the computational burden in the subsequent alignment step). We performed multiple alignment [69] for each set of 25,000 sequences with relatively strict gap penalties to prevent exceedingly large alignments (-25 and -10 gap opening and extension penalties, respectively). We finally mapped the position of the high-ranking *k*-mers to the alignments. The position is the range in which the *k*-mer spans in the multiple alignment, including the presence of gaps.

### Identification of important node activations

We calculated sequence activations for each body site in a similar manner as described above. To obtain sequence activations, we calculated the outer product between all sequence embeddings and the sparse set of lasso regression coefficients: *α*_*r*_ = *ε*_*r*_ ⊗ *β*, where, for a corpus of R sequences, *α*_*r*_ is an *R* × *J* matrix of sequence activations for J body sites, *ε*_*r*_ is an *R* × *d* matrix of sequence embeddings, and *β* is a *d* × *J* matrix of regression coefficients. Then, for a given sample, we summed each sequence activation *α*_*r*_ to obtain a cumulative sum for all sequence activations through sequence activation *α*_*r*_.

## Supporting information

S1 Appendix Contains additional information regarding the following: (1) expands upon the results found in the section that described how *k*-mers associated with Lachnospiraceae genera were associated with the skin body site and sequence regions by providing additional information at the phylum level and for fecal and tongue body sites; (2) compares results to a similar embedding algorithm, doc2vec; and (3) fully describes the parameter selection procedure and the results used to justify the selected parameterization.
(PDF)

## References

[1] Gevers D, Kugathasan S, Denson L, Vázquez-Baeza Y, Van Treuren W, Ren B, et al The Treatment-Naive Microbiome in New-Onset Crohn’s Disease. Cell Host & Microbe. 2014;15(3):382–392. 10.1016/j.chom.2014.02.005 24629344 PMC4059512

[2] Schmidt BL, Kuczynski J, Bhattacharya A, Huey B, Corby PM, Queiroz EL, et al Changes in abundance of oral microbiota associated with oral cancer. PLoS One. 2014;9(6):e98741 10.1371/journal.pone.0098741 24887397 PMC4041887

[3] Woloszynek S, Pastor S, Mell JC, Nandi N, Sokhansanj B, Rosen GL. Engineering Human Microbiota: Influencing Cellular and Community Dynamics for Therapeutic Applications. International Review of Cell and Molecular Biology. 2016;324:67–124. 10.1016/bs.ircmb.2016.01.003 27017007

[4] Henry S, Baudoin E, López-Gutiérrez JC, Martin-Laurent F, Brauman A, Philippot L. Quantification of denitrifying bacteria in soils by nirK gene targeted real-time PCR. Journal of Microbiological Methods. 2004;59(3):327–335. 10.1016/j.mimet.2004.07.002 15488276

[5] Okano Y, Hristova KR, Leutenegger CM, Jackson LE, Denison RF, Gebreyesus B, et al Application of Real-Time PCR to Study Effects of Ammonium on Population Size of Ammonia-Oxidizing Bacteria in Soil. Applied and Environmental Microbiology. 2004;70(2):1008–1016. 10.1128/AEM.70.2.1008-1016.2004 14766583 PMC348910

[6] Sunagawa S, Coelho LP, Chaffron S, Kultima JR, Labadie K, Salazar G, et al Structure and function of the global ocean microbiome. Science. 2015;348 (6237). 10.1126/science.1261359 25999513

[7] de Vos WM, De Vos EAJ. Role of the intestinal microbiome in health and disease: From correlation to causation. Nutrition Reviews. 2012;70(SUPPL. 1):45–56. 10.1111/j.1753-4887.2012.00505.x 22861807

[8] Ni J, Wu GD, Albenberg L, Tomov VT. Gut microbiota and IBD: Causation or correlation?; 2017. 10.1038/nrgastro.2017.88 PMC5880536 28743984

[9] Saraswati S, Sitaraman R. Aging and the human gut microbiota-from correlation to causality. Frontiers in Microbiology. 2015;5(January):1–4. 10.3389/fmicb.2014.00764 PMC4290626 25628610

[10] Harley ITW, Karp CL. Obesity and the gut microbiome: Striving for causality; 2012. 10.1016/j.molmet.2012.07.002 PMC3757651 24024115

[11] Caporaso J, Kuczynski J, Stombaugh J, Bittinger K, Bushman. QIIME allows analysis of high-throughput community sequencing data. Nature Methods. 2012;7:335–336. 10.1038/nmeth.f.303 PMC3156573 20383131

[12] Callahan BJ, McMurdie PJ, Rosen MJ, Han AW, Johnson AJA, Holmes SP. DADA2: High-resolution sample inference from Illumina amplicon data. Nature Methods. 2016;13(7):581–583. 10.1038/nmeth.3869 27214047 PMC4927377

[13] Nguyen NP, Warnow T, Pop M, White B. A perspective on 16S rRNA operational taxonomic unit clustering using sequence similarity. Npj Biofilms And Microbiomes. 2016;2:16004 EP –. 10.1038/npjbiofilms.2016.4 28721243 PMC5515256

[14] Callahan BJ, McMurdie PJ, Holmes SP. Exact sequence variants should replace operational taxonomic units in marker-gene data analysis. The Isme Journal. 2017;11:2639 EP –. 10.1038/ismej.2017.119 28731476 PMC5702726

[15] Mysara M, Vandamme P, Props R, Kerckhof FM, Leys N, Boon N, et al Reconciliation between operational taxonomic units and species boundaries. FEMS Microbiology Ecology. 2017;93(4):fix029 10.1093/femsec/fix029 PMC5812548 28334218

[16] Edgar RC. Updating the 97 Bioinformatics. 2018;34(14):2371–2375. 10.1093/bioinformatics/bty113 29506021

[17] Lan Y, Morrison JC, Hershberg R, Rosen GL. POGO-DB—a database of pairwise-comparisons of genomes and conserved orthologous genes. Nucleic Acids Research. 2014;42(D1):D625–D632. 10.1093/nar/gkt1094 24198250 PMC3964953

[18] McDonald D, Hyde ER, Debelius JW, Morton JT, Gonzalez A, Ackermann G, et al. American Gut: an Open Platform for Citizen-Science Microbiome Research. bioRxiv. 2018;. 10.1128/mSystems.00031-18 PMC5954204 29795809

[19] Nelson MC, Morrison HG, Benjamino J, Grim SL, Graf J. Analysis, optimization and verification of illumina-generated 16s rRNA gene amplicon surveys. PLoS ONE. 2014;. 10.1371/journal.pone.0094249 PMC3983156 24722003

[20] Golob JL, Margolis E, Hoffman NG, Fredricks DN. Evaluating the accuracy of amplicon-based microbiome computational pipelines on simulated human gut microbial communities. BMC Bioinformatics. 2017;. 10.1186/s12859-017-1690-0 28558684 PMC5450146

[21] Ng P. dna2vec: Consistent vector representations of variable-length k-mers. 2017; p. 1–10.

[22] Choong ACH, Lee NK. Evaluation of Convolutionary Neural Networks Modeling of DNA Sequences using Ordinal versus one-hot Encoding Method. bioRxiv. 2017; p. 186965.

[23] Voss RH, Hartmann RK, Lippmann C, Alexander C, Jahn O, Erdmann VA. Sequence of the tufA gene encoding elongation factor EF-Tu from Thermus aquaticus and overproduction of the protein in Escherichia coli.; 1992. 10.1111/j.1432-1033.1992.tb17115.x 1499561

[24] Bengio Y, Senécal JS. Adaptive importance sampling to accelerate training of a neural probabilistic language model. IEEE Transactions on Neural Networks. 2008;19(4):713–722. 10.1109/TNN.2007.912312 18390314

[25] Wood DE, Salzberg SL. Kraken: ultrafast metagenomic sequence classification using exact alignments. Genome biology. 2014;15(3):R46 10.1186/gb-2014-15-3-r46 24580807 PMC4053813

[26] DeSantis TZ, Hugenholtz P, Larsen N, Rojas M, Brodie EL, Keller K, et al Greengenes, a Chimera-Checked 16S rRNA Gene Database and Workbench Compatible with ARB. Applied and Environmental Microbiology. 2006;72(7):5069–5072. 10.1128/AEM.03006-05 16820507 PMC1489311

[27] Mikolov T, Chen K, Corrado G, Dean J. Efficient Estimation of Word Representations in Vector Space Arxiv. 2013; p. 1–12.

[28] Johnson R, Zhang T. Supervised and Semi-Supervised Text Categorization using LSTM for Region Embeddings. 2016;48.

[29] Min X, Zeng W, Chen N, Chen T, Jiang R. Chromatin accessibility prediction via convolutional long short-term memory networks with k-mer embedding In: Bioinformatics. vol. 33; 2017 p. i92–i101. 28881969 10.1093/bioinformatics/btx234 PMC5870572

[30] Pandey C, Ibrahim Z, Wu H, Iqbal E, Dobson R. Improving RNN with atention and embedding for adverse drug reactions. In: ACM International Conference Proceeding Series. vol. Part F1286; 2017. p. 67–71. Available from: https://www.scopus.com/inward/record.uri?eid=2-s2.0-85025443946&doi=10.1145%2F3079452.3079501&partnerID=40&md5=43776389473a0b5f35b7fe71007f564d.

[31] Bengio Y, {LeCun} Y, Lecun Y. Scaling Learning Algorithms towards AI. Large Scale Kernel Machines. 2007;(1):321–360.

[32] Le Q, Mikolov T. Distributed Representations of Sentences and Documents. International Conference on Machine Learning—ICML 2014. 2014;32:1188–1196.

[33] Bahdanau D, Bosc T, Jastrzȩbski S, Grefenstette E, Vincent P, Bengio Y. Learning to Compute Word Embeddings on the Fly. 2017; (2015).

[34] Pennington J, Socher R, Manning C. Glove: Global Vectors for Word Representation. In: Proceedings of the 2014 Conference on Empirical Methods in Natural Language Processing (EMNLP); 2014. p. 1532–1543. Available from: http://aclweb.org/anthology/D14-1162.

[35] Li J, Chen X, Hovy E, Jurafsky D. Visualizing and Understanding Neural Models in NLP. 2015;.

[36] Athiwaratkun B, Wilson AG. Multimodal Word Distributions. 2017;.

[37] Asgari E, Mofrad MRK. Continuous distributed representation of biological sequences for deep proteomics and genomics. 2015;10(11). 10.1371/journal.pone.0141287 PMC4640716 26555596

[38] Drozd A, Gladkova A, Matsuoka S. Word Embeddings, Analogies, and Machine Learning: Beyond King—M an + W oman = Queen. In: Proceedings of COLING 2016; 2016. p. 3519–3530.

[39] Arora S, Liang Y, Ma T. A simple but tough to beat baseline for sentence embeddings Iclr. 2017; p. 1–14.

[40] Tenenbaum D. KEGGREST: Client-side REST access to KEGG; 2018.

[41] Van Der Maaten LJP, Hinton GE. Visualizing high-dimensional data using t-sne. Journal of Machine Learning Research. 2008;9:2579–2605.

[42] Rognes T, Flouri T, Nichols B, Quince C, Mahé F. VSEARCH: a versatile open source tool for metagenomics. PeerJ. 2016;4:e2584 10.7717/peerj.2584 27781170 PMC5075697

[43] Lloyd SP. Least Squares Quantization in PCM. IEEE Transactions on Information Theory. 1982;28(2):129–137. 10.1109/TIT.1982.1056489

[44] Friedman J, Hastie T, Tibshirani R. Regularization Paths for Generalized Linear Models via Coordinate Descent. Journal of Statistical Software. 2010;33(1). 10.18637/jss.v033.i01 20808728 PMC2929880

[45] Tibshirani R. Regression Selection and Shrinkage via the Lasso; 1996 Available from: http://citeseer.ist.psu.edu/viewdoc/summary?doi=10.1.1.35.7574.

[46] Gloor GB, Reid G. Compositional analysis: a valid approach to analyze microbiome high throughput sequencing data. Canadian Journal of Microbiology. 2016;703(April):2015–0821. 10.1139/cjm-2015-0821 27314511

[47] Kumar MS, Slud EV, Okrah K, Hicks SC, Hannenhalli S, Corrada Bravo H. Analysis And Correction Of Compositional Bias In Sparse Sequencing Count Data. bioRxiv. 2017; p. 1–34. 10.1186/s12864-018-5160-5 PMC6219007 30400812

[48] Krakovna V, Doshi-Velez F. Increasing the Interpretability of Recurrent Neural Networks Using Hidden Markov Models. ArXiv. 2016;(Whi):2012–2017.

[49] Lanchantin J, Singh R, Wang B, Qi Y. Deep Motif Dashboard: Visualizing and Understanding Genomic Sequences Using Deep Neural Networks. arXiv. 2016; p. 1–11. 10.1142/9789813207813_0025 PMC5787355 27896980

[50] Alain G, Bengio Y. Understanding intermediate layers using linear classifier probes. 2016;.

[51] Samek W. Methods for Interpreting and Understanding Deep Neural Networks;.

[52] Kim B, Shah J, Doshi-Velez F. Mind the Gap: A Generative Approach to Interpretable Feature Selection and Extraction. Nips. 2015; p. 1–9.

[53] Oh J, Conlan S, Polley EC, Segre JA, Kong HH. Shifts in human skin and nares microbiota of healthy children and adults. Genome Medicine. 2012;4(10). 10.1186/gm378 23050952 PMC3580446

[54] Byrd AL, Belkaid Y, Segre JA. The human skin microbiome; 2018. 10.1038/nrmicro.2017.157 29332945

[55] Ling Z, Liu X, Cheng Y, Jiang X, Jiang H, Wang Y, et al Decreased Diversity of the Oral Microbiota of Patients with Hepatitis B Virus-Induced Chronic Liver Disease: A Pilot Project. Scientific Reports. 2015;5 10.1038/srep17098 PMC4660595 26606973

[56] Chen H, Jiang W. Application of high-throughput sequencing in understanding human oral microbiome related with health and disease. Frontiers in Microbiology. 2014;5(SEP). 10.3389/fmicb.2014.00508 PMC4195358 25352835

[57] Goodrich JK, Waters JL, Poole AC, Sutter JL, Koren O, Blekhman R, et al Human genetics shape the gut microbiome. Cell. 2014;159(4):789–799. 10.1016/j.cell.2014.09.053 25417156 PMC4255478

[58] Dulal S, Keku TO. Gut microbiome and colorectal adenomas. Cancer J. 2014;20(3):225–231. 10.1097/PPO.0000000000000050 24855012 PMC4589167

[59] Graessler J, Qin Y, Zhong H, Zhang J, Licinio J, Wong ML, et al Metagenomic sequencing of the human gut microbiome before and after bariatric surgery in obese patients with type 2 diabetes: correlation with inflammatory and metabolic parameters. The Pharmacogenomics Journal. 2012;(October 2012):514–522. 10.1038/tpj.2012.43 23032991

[60] Crielaard W, Zaura E, Schuller AA, Huse SM, Montijn RC, Keijser BJF. Exploring the oral microbiota of children at various developmental stages of their dentition in the relation to their oral health. BMC Medical Genomics. 2011;4 10.1186/1755-8794-4-22 21371338 PMC3058002

[61] Sampaio-Maia B, Monteiro-Silva F. Acquisition and maturation of oral microbiome throughout childhood: An update. Dental research journal. 2014;11(3):291–301. 25097637 PMC4119360

[62] Ching T, Himmelstein DS, Beaulieu-Jones BK, Kalinin AA, Do BT, Way GP, et al Opportunities and obstacles for deep learning in biology and medicine. J R Soc Interface. 2018;15(141). 10.1098/rsif.2017.0387 29618526 PMC5938574

[63] DeSantis TZ, Hugenholtz P, Larsen N, Rojas M, Brodie EL, Keller K, et al Greengenes, a chimera-checked 16S rRNA gene database and workbench compatible with ARB. Applied and Environmental Microbiology. 2006;72(7):5069–5072. 10.1128/AEM.03006-05 16820507 PMC1489311

[64] Rehurek R, Sojka P. Software Framework for Topic Modelling with Large Corpora. Proceedings of the LREC 2010 Workshop on New Challenges for NLP Frameworks. 2010; p. 45–50.

[65] Pennington J, Socher R, Manning CD. GloVe: Global Vectors for Word Representation In: Empirical Methods in Natural Language Processing (EMNLP); 2014 p. 1532–1543. Available from: http://www.aclweb.org/anthology/D14-1162.

[66] Asgari E, Garakani K, Mofrad MRK. A New Approach for Scalable Analysis of Microbial Communities. CoRR. 2015;abs/1512.00397.

[67] Asgari E, Garakani K, McHardy AC, Mofrad MRK. MicroPheno: Predicting environments and host phenotypes from 16S rRNA gene sequencing using a k-mer based representation of shallow sub-samples. bioRxiv. 2018;. 10.1093/bioinformatics/bty296 PMC6022683 29950008

[68] Wang Q, Garrity GM, Tiedje JM, Cole JR. Naïve Bayesian classifier for rapid assignment of rRNA sequences into the new bacterial taxonomy. Applied and Environmental Microbiology. 2007;73(16):5261–5267. 10.1128/AEM.00062-07 17586664 PMC1950982

[69] Wright ES. DECIPHER: Harnessing local sequence context to improve protein multiple sequence alignment. BMC Bioinformatics. 2015;16(1). 10.1186/s12859-015-0749-z 26445311 PMC4595117

