# Supplementary material for: 16S rRNA sequence embeddings: Meaningful numeric feature representations of nucleotide sequences that are convenient for downstream analyses
Source: PLoS Comput Biol. 2019 Feb 26;15(2):e1006721. doi: 10.1371/journal.pcbi.1006721 (PMC6407789; doi:10.1371/journal.pcbi.1006721)
Supplement: S1 Appendix — Contains additional information regarding the following: (1) expands upon the results found in the section that described how k-mers associated with Lachnospiraceae genera were associated with the skin body site and sequence regions by providing additional information at the phylum level and for fecal and tongue body sites; (2) compares results to a similar embedding algorithm, doc2vec; and (3) fully describes the parameter selection procedure and the results used to justify the selected parameterization. (PDF) [file pcbi.1006721.s001.pdf]

16S rRNA sequence embeddings: Meaningful numeric feature representations of nucleotide sequences that are convenient for downstream analyses

Stephen Woloszynek[1], Zhengqiao Zhao[1], Jian Chen[2], Gail L. Rosen[1]*

1 Department of Electrical and Computer Engineering, Drexel University, Philadelphia, PA, USA
2 Department of Computer Science and Engineering, State University of New York at Buffalo, Buffalo, NY, USA

* glr26@drexel.edu

S1 Appendix

Results and Discussion

Specific *k*-mer profiles are associated with different body sites and these *k*-mers favor specific regions of the 16S rRNA gene

Our objectives were to identify (1) k-mers that strongly influence the performance during sample-level (body site) classification and (2) characteristics of the sequences that contain these k-mers, such as the sequence's taxonomic classification vis-á-vis the neighborhood in which important k-mers occur. We again obtained the sparse set of regression coefficients that were estimated via lasso to classify body site using sample embeddings of the American Gut data. We calculated k-mer activations (the linear combination of the lasso regression coefficients and the k-mer embeddings) and then selected the top-1000 k-mers with largest activations for classifying skin, tongue, or fecal body sites. These k-mers, when present in a sequence, have the single greatest influence on the classification outcome for a given sample, although it should be noted that the final classification decision is predominantly driven by high-frequency (after weighting) k-mers with relatively large activations.

Here we focus on the classification of skin samples. To further characterize the k-mer profiles, we associated high-activation k-mers with the neighborhood of their read from which they originated. For each set of reads that contained the high-activation k-mers, we randomly sampled 25,000 reads and performed multiple alignment. Fig A shows the relative regions (in their multiple alignment) in which these k-mers typically originated. Five 10-mers that were associated with skin spanned positions 116 to 146 in the multiple alignment: CGTTTTAAGT, GGTTACTCAA, GTTACTCAAG, TTACTCAAGT, and TACTCAAGTC. These 10-mers belonged to Proteobacteria and were notable for occurring closer to the beginning of the multiple alignment. Downstream, three 10-mers from reads classified as Firmicutes spanned positions 127 to 392: TTTTAAGCCT, TTAAGCCTGA, and GGGGGTCATT. Finally, three additional 10-mers from reads classified as Actinobacteria spanned positions 191 to 361: TTGTGTAATA, GGTCTGCAGG, TCTGCAGGCG. For fecal- and tongue-associated k-mers, most mapped to mid-to-downstream regions in their respective multiple alignments, at approximately positions 200 through 550. Gut-associated k-mers were most abundant between positions 300 and 500 and mostly mapped to reads with Firmicutes (40 unique k-mers across 64,236 of 181,950 total reads in this region) and

Bacteroidetes (23 unique k-mers across 92,545 reads). Lastly, tongue-associated k-mers were the most downstream of the three body sites, favoring nucleotide positions 300 to 500 and mapping mainly to reads labeled as Firmicutes (72 unique k-mers across 117,463 of 324,704 total reads in this region) and Proteobacteria (83 unique k-mers across 65,535 reads).

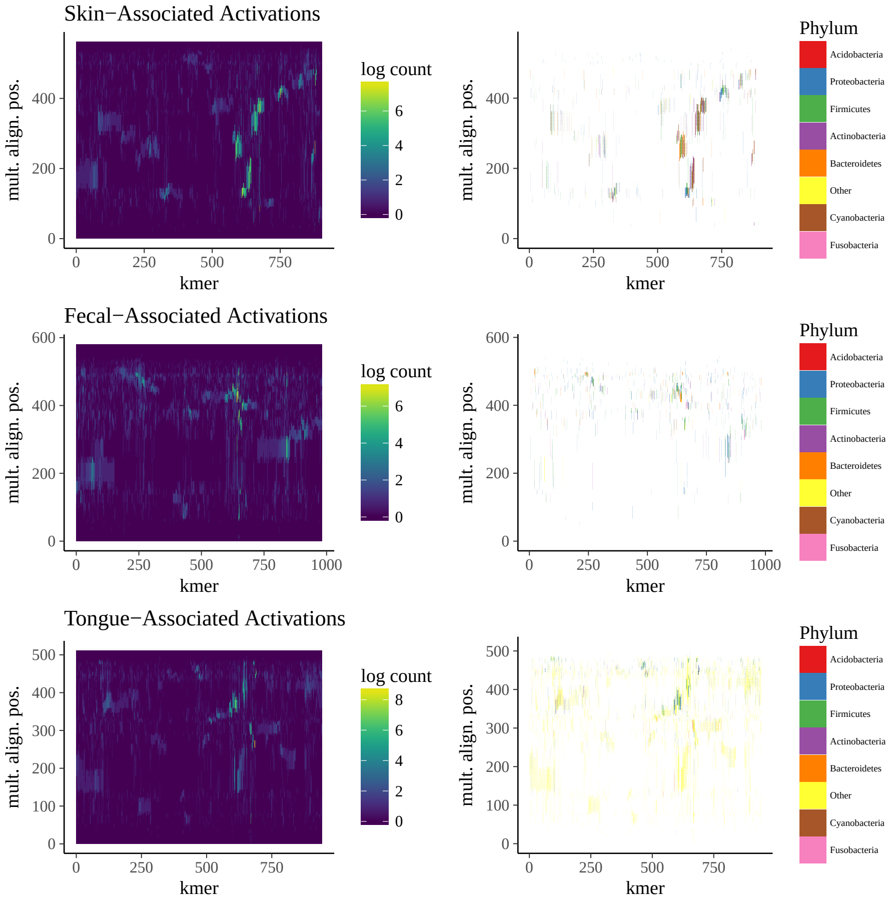

Fig A. Regions within reads in which high-activation *k*-mers mapped. The top-1000 k-mers with the largest activations (the linear combination of the k-mer embedding and regression coefficients obtained via lass) for a given body site were identified. A random sample of 25,000 reads (from skin samples) containing these k-mers underwent multiple alignment. Shown are the relative positions of each set of 1000-kmers in the the three multiple alignments. The position of a given k-mer in the alignment spans its entire starting and ending position, including gaps. The frequency in which k-mers mapped to positions (left column) in the multiple alignment were quantified (y-axis). The order of the k-mers in the heatmap (x-axis) was obtained via hierarchical clustering (Ward's method) on Bray-Curtis distances. The low log counts at the flanks of the alignment are due to terminal gaps. For each alignment heatmap, the phylum that most frequently occurred at a given alignment position for a given k-mer is colored (right column).

It should be noted that this approach is limited by the taxonomic classifications of the reads. American Gut reads are short; after pre-processing for quality, they were only 125 nucleotides long. Consequently, very few reads received lower-level taxonomic

classifications with high confidence. For example, of the 241,006 reads in skin samples, only 39.4% of reads received genus classifications (398 unique genera) with greater than 50% bootstrap confidence and only 31.1% (236 unique genera) at 80% bootstrap confidence. This hinders the extent in which meaning can be captured from the results, particularly at lower taxonomic levels. Considering there is substantial intra-phylum read variation, particularly in variable regions of the 16S rRNA gene, focusing on genera that share a family, for example, may present interesting patterns as to how specific k-mers, associated with specific taxa, favor specific regions in the 16S rRNA gene.

The optimal parameterization utilized 10-mers, was denoised, and received moderate *k*-mer down-weighting

The model parameterization used to generate the results described above was obtained by sweeping over various word2vec parameterizations, as well as exploring different strategies for embedding sequences and samples. Specifically, we trained word2vec models that differed in terms of the dimensionality of the embedding (the dimensionality of the hidden layer; 64, 128, 256); the length of the k-mers to be embedded (6, 10); the threshold in which high frequency k-mers are down-sampled during training (0.0001, 0.000001); the number of negative samples for training (10, 20); and the width of the context window during training (20, 50). When generating sequence and sample embeddings, we varied the degree in which k-mers were down-weighted with respect to their frequency during embedding (0.0, 0.00001, 0.0000001) and whether the projection to the first principle component was removed. We then assessed the effect varying these parameters had on the embedding in two ways: (1) the sign-rank correspondence of a sequence's nearest neighbor obtained via alignment (which we treat as the gold standard) versus its nearest neighbor obtained via cosine similarity in the embedding space and (2) the similarity (adjusted rand index (ARI)) between sequences of a certain taxonomic class (*e.g.*, Bacteroidetes or *Escherichia* at the phylum or genus levels, respectively) and clusters generated via unsupervised clustering. For both approaches, we regressed their score (sign-rank correspondence and ARI) against the parameter categories.

Fig B shows the regression coefficients for each regression. For the alignment approach (top), a regression was performed for each subset of sequences with similar alignment sequence identity, whereas for the clustering approach (bottom), a regression was performed at each taxonomic level. Together, the analyses suggest that as the similarity between sequences increases (in terms alignment similarity or taxonomic level), performance improved with a moderate degree of down-weighting of high frequency k-mers, removal of the projection to the first principle component after embedding sequences from k-mers, and a large context window during training. The number of negative samples and dimensionality of the embedding space seemingly had little effect.

Large context window sizes improving performance was simply due to k-mers being generated by a sliding window. A shift of the window by one position in either direction captures very little of the k-mer's neighborhood; these neighbors differ by only one nucleotide. Thus, to capture meaningful information of a k-mer's context, a large context window is required. The improvement due to removing the projection to the first principle component is consistent with [1], which reported a 10% improvement in textual similarity tasks. They concluded that it acts as a form of denoising.

The length of the k-mers affected the alignment and clustering approaches differently. Larger k-mers (10-mers) improved clustering performance, albeit slightly ($\beta_{genus}, = 0.014, \beta_{species}, = 0.005$), but only when similarity was high among sequences (genus and species-level), whereas smaller k-mers (6-mers) increased the correspondence of the embedding space with alignment, but again only for highly similar sequences. This disagreement may be in part due to there being few genus and species sequence

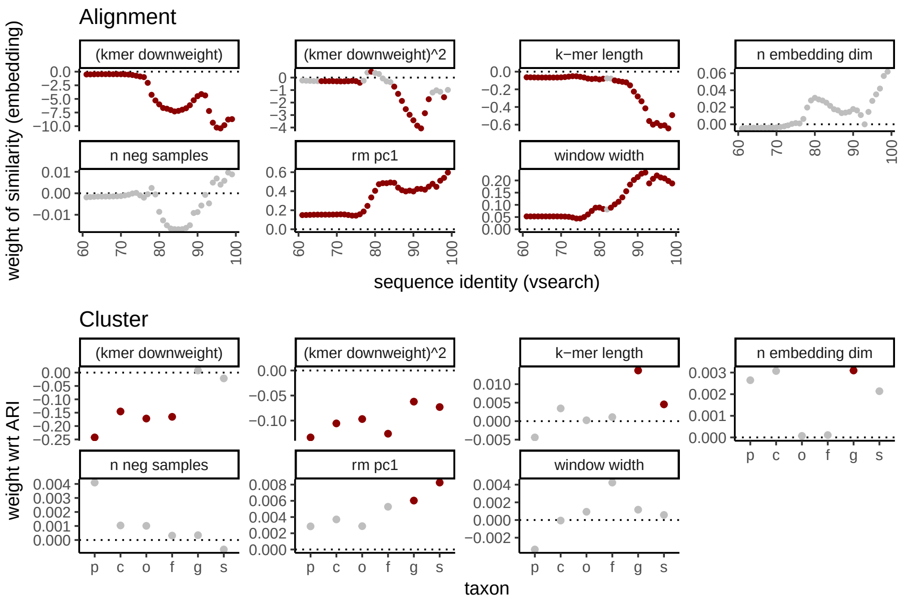

Fig B. Benchmarking results to determine an optimal word2vec parameterization and general embedding procedure. Top: Benchmarking results with respect to global alignment using KEGG 16S sequences. Each point is a regression coefficient from a Gamma regression model, where the dependent variable was the agreement (sign-rank correspondence) between cosine similarity in the embedding space and global alignment identity using v-search. Significant FDR-adjusted p-values are colored red when $p < 0.1$. A model was fit that included all variables shown for each set of sequences that had similar sequence identity (x-axis), which was determined via v-search global alignment. k-mer down-weighting during training and embedding were grouped into a single ranked covariate and fit with a second degree polynomial. Bottom: Benchmarking results via K-means clustering. Clustering of sequence embeddings and Gamma regression took place within a given taxonomic level (x-axis), where the dependent variable was ARI. P-values were FDR-adjusted, where significant regression coefficients are colored sequence when $p < 0.1$.

labels. Also, because the number of possible 10-mers (4^{10}) is much greater that of 6-mers (4^6), the likelihood that a given query 10-mer was not present during training, and hence does not have an embedding representation, is greater. This adds yet additional variability due to small sample size in that a 10-mer sequence embedding may have had contribution from few 10-mers relative to a 6-mer sequence embedding. This may also explain why 6-mers yield better performance for the alignment analysis: lacking a key k-mer embedding that distinguishes two highly similar sequences is more likely to occur for larger k-mers. Thus, this result may be different with better training data that covers close to all k-mer possibilities in a given query dataset.

Doc2vec performed worse than the optimal sequence embedding

To generate sample embeddings, we must combine all k-mer embeddings that encompass all sequences belonging to a given sample. Our method is based on the word2vec model (as well as sentence embedding post-processing) that embeds individual words (based on their context) into vector representations. Alternatively, [2] proposed to embed a whole paragraph, document, or sentence into a vector representation, such that no longer must

one average or sum the word embeddings together to obtain a paragraph, document, sentence embedding. Similar to word vectors, the document vectors are also learned by training a neural network to predict the word given its context. In addition to using context words, doc2vec also treats the document label (from which the context is obtained) as a word. This document label will be embedded by the neural network to produce a document vector that is an abstraction of the document.

We benchmarked the efficacy of doc2vec at embedding DNA sequences. We used the gensim implementation to train the doc2vec sequence embedding using sequences from the GreenGenes database. We then embedded the KEGG 16S sequences and performed clustering using K-means in the same fashion described above using our embedding approach. The doc2vec parameters chosen were set to the optimal set of word2vec parameters we identified via our alignment and clustering tests. Table A compares the clustering performance of doc2vec and our embedding approach. Doc2vec struggled to resolve sequences in terms of their taxonomy. At the species level, doc2vec had a poor homogeneity score of 0.54; that is, sequences that are close in the doc2vec embedding space were not necessarily similar to each other.

Table A. Clustering analysis of KEGG doc2vec sequence embeddings

Taxon	K	P	C	O	F	G	S
# of Taxa	2	35	77	143	281	697	385
K	2	35	77	143	281	697	385
Homogeneity	0.00	0.14	0.21	0.25	0.35	0.47	0.54
Completeness	0.00	0.07	0.12	0.18	0.27	0.40	0.49
ARI	0.01	0.01	0.02	0.02	0.02	0.02	0.04
AMI	0.00	0.06	0.10	0.13	0.16	0.15	0.20
NMI	0.00	0.10	0.16	0.21	0.31	0.44	0.52

Clustering the embeddings yield higher fidelity clusters for species than for higher taxonomic levels

Table B. Clustering analysis of KEGG sequence 6-mer frequency table

Taxon	K	P	C	O	F	G	S
# of Taxa	2	35	77	143	281	697	385
K	2	35	77	143	281	697	385
Homogeneity	0.08	0.87	0.93	0.94	0.95	0.97	0.98
Completeness	0.01	0.38	0.50	0.65	0.75	0.80	0.90
ARI	-0.02	0.14	0.15	0.25	0.26	0.33	0.58
AMI	0.01	0.38	0.49	0.63	0.70	0.71	0.84
NMI	0.03	0.58	0.68	0.78	0.84	0.88	0.94

Materials and Methods

Parameter benchmarking via alignment

The KEGG sequences underwent pairwise alignment via v-search. Weak-alignments with pairwise sequence identity below 0.6 were discarded, where sequence identity is defined as $100 \times$ (matching columns)/(alignment length-terminal gaps). Then, for the embedding of given sequence i, we calculated its cosine similarity to all other sequence embeddings $\neg i$

We calculated the rank difference between the vector of alignment identities, $\mathrm{id}(\mathrm{sequence}i, \mathrm{sequence}\neg i)$, and the vector of cosine similarities, $\mathrm{cosim}(\mathrm{embedding}i, \mathrm{embedding}\neg i)$, as follows. Both vectors were independently ranked (ties were replaced by their average value). Sequence pairs $(i, \neg i)$ were then binned based on their v-search sequence identity. Then, within each bin, we computed $\sum |\mathrm{rank}_{cosim} - \mathrm{rank}_{id}|/N_{bin}$, where N_{bin} is the number of sequence pairs $(i, \neg i)$ falling into a bin. Performing the comparison within bin allowed us to assess whether the embedding performance varied as a function of sequence similarity.

To determine the effect different parameterizations had on the rank difference between alignment and sequence embedding cosine similarity, we performed Gamma regression (log-link) with the various parameters as independent variables and the rank difference score as the dependent variable. Embedding dimensionality was converted into an ordinal covariate $(1, 2, 3)$. Down-sampling for training and embedding were combined into an ordinal covariate of length 5 $(1, \ldots, 5)$, where a larger rank suggests more down-sampling of high-frequency k-mers. A second-degree polynomial was used to account for the nonlinear association down-sampling had with the rank difference score. All other variables were treated as binary covariates. These included the width of the context window, length of k-mers, and whether the embedding was denoised (removal of the projection to the first principal component). Regressions were performed for each model parameterization within each sequence identity bin, followed by false discovery rate (FDR) adjustment to account for multiple comparisons. Regression coefficients were considered significant when the adjusted p-value was less than 0.1.

Parameter benchmarking via clustering

To benchmark different parameterizations and select the best model, for each set of parameters, we clustered KEGG sequences based on their sequence embeddings by K-means [3]. The free parameter in the algorithm, K, was chosen according to the total number of unique taxa within a taxonomic level. The naive Bayes RDP classifier implemented in QIIME (assign_taxonomy.py, default settings) was used to label the taxonomy of the KEGG sequences. sequences lacking a classification at a given taxonomic level were omitted. We used clustering evaluation metrics to evaluate the performance of clustering results. These evaluation results can be used to evaluate the embedding space in its ability to resolve sequences from different taxa and hence group sequences from the same taxon. These metrics include Homogeneity, Completeness, ARI, AMI, and NMI for each model and each taxon level.

Homogeneity and Completeness are two complementary ways to evaluate the clustering performance [4]. A clustering result satisfies homogeneity if all of its clusters contain only data points that are members of a single class. A clustering result satisfies completeness if all the data points that are members of a given class are elements of the same cluster [4]. Homogeneity is defined as:

$$h = \begin{cases} 1, & H(C,K) = 0 \\ 1 - \dfrac{-\sum\limits_{k=1}^{|K|}\sum\limits_{c=1}^{|C|} \frac{a_{ck}}{N} log \frac{a_{ck}}{\sum_{c=1}^{|C|} a_{ck}}}{-\sum\limits_{c=1}^{|C|} \frac{\sum_{k=1}^{|K|} a_{ck}}{n} log \frac{\sum_{k=1}^{|K|} a_{ck}}{n}}, & H(C,K) \neq 0 \end{cases} \tag{1}$$

where a_{ck} is the number of instances that are members of class c as well as the elements of cluster k; N is the total number of instances and n; $|C|$ is the number of true classes; and $|K|$ is the number of predicted clusters.

Normalized Mutual Information (NMI) measures the quality of the clustering against the number of clusters by entropy. This metric can penalizes large numbers of clusters.

$$\mathrm{NMI}(\Omega, C) = \frac{2I(\Omega; C)}{H(\Omega) + H(C)} \tag{2}$$

Both Adjusted Rand index (ARI) and Adjusted Mutual Information (AMI) measure the consistency of the predicted clusters with respect to the ideal classes [5]. It is also adjusted based on the size of clusters so that the adverse affect of unbalanced classes is relieved.

$$\mathrm{ARI} = \frac{RI - E(RI)}{max(RI) - E(RI)} \tag{3}$$

$$\mathrm{AMI}(\Omega, C) = \frac{MI(\Omega; C) - E(MI(\Omega; C))}{max(H(\Omega), H(C)) - E(MI(\Omega; C))} \tag{4}$$

To determine the effect different parameterizations had on ARI, we performed the same Gamma regression (log-link) model described above, but with ARI as the dependent variable. Regressions were performed for each model parameterization within each taxonomic level, followed by FDR-adjustment to account for multiple comparisons. Regression coefficients were considered significant when the adjusted p-value was less than 0.1

Doc2vec training

Generation of sequence embedding using doc2vec was performed with gensim. Parameters were set to be analogous with the word2vec models to be compared; otherwise, default parameters were selected.

References

1. Arora S, Liang Y, Ma T. A simple but tough to beat baseline for sentence embeddings. Iclr. 2017; p. 1–14.

2. Le Q, Mikolov T. Distributed Representations of Sentences and Documents. International Conference on Machine Learning - ICML 2014. 2014;32:1188–1196. doi:10.1145/2740908.2742760.

3. Lloyd SP. Least Squares Quantization in PCM. IEEE Transactions on Information Theory. 1982;28(2):129–137. doi:10.1109/TIT.1982.1056489.

4. Rosenberg A, Hirschberg J. V-measure: A conditional entropy-based external cluster evaluation measure. In: Proceedings of the Joint Conference on Empirical Methods in Natural Language Processing and Computational Natural Language (EMNLP-CoNLL'07). vol. 1; 2007. p. 410–420. Available from: `http://acl.ldc.upenn.edu/D/D07/D07-1043.pdf`.

5. Manning CD, Raghavan P. An Introduction to Information Retrieval. In: Online; 2009. p. 1. Available from: `http://dspace.cusat.ac.in/dspace/handle/123456789/2538`.
